# Association of Race/Ethnicity-Specific Changes in Antihypertensive Medication Classes Initiated Among Medicare Beneficiaries With the Eighth Joint National Committee Panel Member Report

**DOI:** 10.1001/jamanetworkopen.2020.25127

**Published:** 2020-11-18

**Authors:** Calvin L. Colvin, Jordan B. King, Suzanne Oparil, Jackson T. Wright, Gbenga Ogedegbe, April Mohanty, Shakia T. Hardy, Lei Huang, Rachel Hess, Paul Muntner, Adam Bress

**Affiliations:** 1 Department of Epidemiology, University of Alabama at Birmingham, Birmingham; 2 Department of Population Health Sciences, University of Utah School of Medicine, Salt Lake City; 3 Institute for Health Research, Kaiser Permanente Colorado, Aurora; 4 Division of Cardiovascular, Disease, Department of Medicine, University of Alabama at Birmingham, Birmingham; 5 Division of Nephrology and Hypertension, Case Western Reserve University, University Hospitals Cleveland Medical Center, Cleveland, Ohio; 6 New York University Grossman School of Medicine, New York; 7 IDEAS Center of Innovation, Veterans Affairs Salt Lake City Health Care System, Salt Lake City, Utah; 8 Department of Internal Medicine, University of Utah School of Medicine, Salt Lake City

## Abstract

**Importance:**

In December 2013, the panel members appointed to the Eighth Joint National Committee on Prevention, Detection, Evaluation, and Treatment of High Blood Pressure (JNC8) published a recommendation that non-Black adults initiate antihypertensive medication with a thiazide-type diuretic, calcium channel blocker, angiotensin-converting enzyme inhibitor (ACEI), or angiotensin receptor blocker (ARB), whereas Black adults initiate treatment with a thiazide-type diuretic or calcium channel blocker. β-Blockers were not recommended as first-line therapy.

**Objective:**

To assess changes in antihypertensive medication classes initiated by race/ethnicity from before to after publication of the JNC8 panel member report.

**Design, Setting, and Participants:**

This serial cross-sectional analysis assessed a 5% sample of Medicare beneficiaries aged 66 years or older who initiated antihypertensive medication between 2011 and 2018, were Black (n = 3303 [8.0%]), White (n = 34 943 [84.5%]), or of other (n = 3094 [7.5%]) race/ethnicity, and did not have compelling indications for specific antihypertensive medication classes.

**Exposures:**

Calendar year and period after vs before publication of the JNC8 panel member report.

**Main Outcomes and Measures:**

The proportion of beneficiaries initiating ACEIs or ARBs and, separately, β-blockers vs other antihypertensive medication classes.

**Results:**

In total, 41 340 Medicare beneficiaries (65% women; mean [SD] age, 75.7 [7.6] years) of Black, White, or other races/ethnicities initiated antihypertensive medication and met the inclusion criteria for the present study. In 2011, 25.2% of Black beneficiaries initiating antihypertensive monotherapy did so with an ACEI or ARB compared with 23.7% in 2018 (*P* = .47 for trend). Among beneficiaries initiating monotherapy, the proportion filling a β-blocker was 20.1% in 2011 and 15.4% in 2018 for White beneficiaries (*P* < .001 for trend), 14.2% in 2011 and 11.1% in 2018 for Black beneficiaries (*P* = .08 for trend), and 11.3% in 2011 and 15.0% in 2018 for beneficiaries of other race/ethnicity (*P* = .40 for trend). After multivariable adjustment and among beneficiaries initiating monotherapy, there was no evidence of a change in the proportion filling an ACEI or ARB before to after publication of the JNC8 panel member report overall (prevalence ratio, 1.00; 95% CI, 0.97-1.03) or in Black vs White beneficiaries (prevalence ratio, 0.96; 95% CI, 0.83-1.12; *P* = .60 for interaction). Among beneficiaries initiating monotherapy, the proportion filling a β-blocker decreased from before to after publication of the JNC8 panel member report (prevalence ratio, 0.89; 95% CI, 0.84-0.93) with no differences across race/ethnicity groups (*P* > .10 for interaction).

**Conclusions and Relevance:**

A substantial proportion of older US adults who initiate antihypertensive medication do so with non–guideline-recommended classes of medication.

## Key Points

**Question:**

Have the initial antihypertensive medication regimens filled by older US adults with hypertension changed following publication of the Eighth Joint National Committee on Prevention, Detection, Evaluation, and Treatment of High Blood Pressure panel member report?

**Findings:**

In this serial cross-sectional study of 41 340 Medicare beneficiaries, there was no statistically significant change in the proportion of Black beneficiaries initiating antihypertensive monotherapy with an angiotensin-converting enzyme inhibitor or angiotensin receptor blocker following publication of the panel members’ report. The proportion initiating β-blocker monotherapy remained high among all race/ethnicity groups.

**Meaning:**

Many older US adults who initiate antihypertensive medication do so with non–guideline-recommended classes of medication.

## Teaser

This serial cross-sectional study assesses whether any race/ethnicity-specific changes occurred from before to after publication of the Eighth Joint National Committee on Prevention, Detection, Evaluation, and Treatment of High Blood Pressure panel member report for the initiation of antihypertensive medication classes in a 5% sample of Medicare beneficiaries aged 66 years or older.

## Introduction

In December 2013, the panel members appointed to the Eighth Joint National Committee on Prevention, Detection, Evaluation, and Treatment of High Blood Pressure (JNC8) published a report with evidence-based treatment recommendations for patients initiating antihypertensive medication.^1^ Both the JNC8 panel member report and the previous seventh (JNC7) guideline recommended thiazide-type diuretics as first-line therapy, regardless of race/ethnicity, among patients without compelling indications for specific classes of antihypertensive medication.^1,2^ However, the JNC8 panel member report differed from the JNC7 guideline by including race/ethnicity-specific recommendations for additional initial medications, either alone or as components of combination therapy. For non-Black adults, the JNC8 panel member report recommended thiazide-type diuretics, calcium channel blockers (CCBs), angiotensin-converting-enzyme inhibitors (ACEIs), or angiotensin receptor blockers (ARBs). Black adults were recommended thiazide-type diuretics or CCBs.^1^ The panel did not recommend β-blockers as first-line therapy in the absence of either coronary heart disease (CHD) or heart failure with reduced ejection fraction.

The race/ethnicity-specific treatment recommendations in the JNC8 panel member report were based on randomized trial data showing that Black adults have smaller blood pressure (BP) and cardiovascular disease (CVD) risk decreases than White adults when treated with an ACEI.^3,4^ Prespecified subgroup analyses of the Antihypertensive and Lipid-Lowering Treatment to Prevent Heart Attack Trial (ALLHAT) found that the risks of stroke, heart failure, and CVD were higher in Black participants randomized to an ACEI vs a thiazide-type diuretic,^3^ and the risk of stroke was higher in Black participants randomized to an ACEI vs a CCB.^4^ No differences were present for White participants. β-Blockers were not recommended for first-line therapy because there is evidence of increased CVD risk with β-blockers compared with ARBs.^5^

The purpose of the present study was to evaluate changes in the initial antihypertensive medication regimens filled between 2011 and 2018 among older US adults who were Black, White, or of other race/ethnicity. In addition, we evaluated changes in the initial classes of antihypertensive medication filled before vs after December 18, 2013, the date the JNC8 panel member report was published.

## Methods

We conducted a serial cross-sectional analysis of a 5% sample of Medicare beneficiaries initiating antihypertensive medication between 2011 and 2018. Medicare is a national program funded by the US federal government that provides health insurance to US adults aged 65 years or older and individuals younger than 65 years who are disabled or have end-stage kidney disease. Deidentified data on Medicare beneficiaries’ inpatient, outpatient, and prescription drug claims were obtained from the Centers for Medicare and Medicaid services. We prepared the current manuscript according to the Strengthening the Reporting of Observational Studies in Epidemiology (STROBE) reporting guideline for cross-sectional studies. The University of Alabama at Birmingham institutional review board approved this study and waived the requirement for obtaining informed consent because the research involved no more than minimal risk to the participants and we are unable to contact beneficiaries because the claims data are deidentified.

Study eligibility was determined for each calendar year, separately (eFigure 1 in the Supplement). We required beneficiaries to have at least 1 pharmacy fill for an antihypertensive medication between 2011 and 2018 (eTable 1 in the Supplement). The date of each beneficiary’s first antihypertensive medication fill in each calendar year defined the index date for that person. Fills for antihypertensive medication were defined using prescription claims in the Medicare Part D file, and we considered all antihypertensive medication classes that beneficiaries filled within 7 days following their index date as part of their initial regimen. Analyses were restricted to beneficiaries 66 years of age or older and younger than 110 years of age on their index date. A minimum age of 66 years was required to allow 365 days prior to the index date (ie, the look-back period) to assess covariates and study eligibility criteria while including beneficiaries with Medicare coverage due to older age vs being disabled or having end-stage kidney disease. To ensure complete data capture, we required beneficiaries to have (1) inpatient (Medicare Part A), outpatient (Medicare Part B), and pharmacy (Medicare Part D) coverage without Medicare Advantage (Medicare Part C) coverage during the look-back period and 7 days of coverage following their index date and (2) continuous residence in the US during this period. To increase the likelihood that the antihypertensive medication was being used to treat hypertension, we required beneficiaries to have at least 2 outpatient claims linked to physician evaluation and management codes 7 days or more apart, with *International Classification of Diseases, Ninth Revision* diagnoses of 401.x (ie, malignant, benign, or unspecified essential hypertension) during the look-back period.

We excluded beneficiaries with more than 1 birth date or death date or, for those who died, any claims after their date of death. We restricted the analysis to beneficiaries initiating treatment by excluding beneficiaries with claims for antihypertensive medication during the look-back period. To evaluate calendar years before and after publication of the JNC8 panel member report,^1^ we excluded beneficiaries whose index date was between December 18 and 31, 2013. Using their index date, we categorized beneficiaries into the year that they initiated antihypertensive medication. Because we were interested in studying treatment initiation among beneficiaries without compelling indications for specific antihypertensive medication classes, we restricted the analysis to those without a history of CHD, diabetes, heart failure, chronic kidney disease, or stroke (eTable 2 in the Supplement)^2^ by using previously published algorithms (eTable 3 in the Supplement). We also excluded beneficiaries with a history of atrial fibrillation or flutter because β-blockers are indicated for heart-rate control in this population.

### Class of Antihypertensive Medication Initiated

We categorized classes of antihypertensive medications as defined in the 2017 American College of Cardiology (ACC)/American Heart Association (AHA) hypertension guideline (eTable 1 in the Supplement).^6^ Beneficiaries were categorized as initiating antihypertensive monotherapy or combination therapy.

### Beneficiary Race/Ethnicity

We used the Medicare beneficiary summary files to define beneficiaries’ race/ethnicity. Medicare obtains data on the race/ethnicity of beneficiaries from the Social Security Administration, which collects voluntarily reported race/ethnicity at the time of application for a Social Security number. Because there were few Asian (n = 951), Hispanic (n = 909), and “other” (n = 1234) race/ethnicity beneficiaries who met the inclusion criteria, we combined these race/ethnicity groups into a single “other race/ethnicity” category for the analysis.

### Covariates

We selected covariates a priori, including beneficiary age at the end of their look-back period, sex, region of residence,^7^ and whether they received care from a cardiologist, endocrinologist, or nephrologist in the look-back period. As markers of socioeconomic status, we identified whether beneficiaries’ Medicare premiums were paid by Medicaid (ie, Medicare-Medicaid dual eligibility) or whether they received a Medicare low-income subsidy during the look-back period and their residence area-level median income (eTable 3 in the Supplement).

### Statistical Analysis

We calculated characteristics of Medicare beneficiaries who initiated antihypertensive medication. Among beneficiaries initiating monotherapy and, separately, combination therapy, we calculated the proportion initiating each antihypertensive class. For beneficiaries initiating antihypertensive combination therapy, we calculated the number and proportion who initiated the 4 most common combinations of dual and triple therapies. All calculations were completed by calendar year and race/ethnicity. We assessed linear trends across calendar years using linear regression for continuous variables and Poisson regression for categorical variables.

We used Poisson regression with robust standard errors to calculate prevalence ratios (PRs) and 95% CIs for initiating antihypertensive medication with an ACEI or ARB vs other antihypertensive classes, for those initiating monotherapy or combination therapy, seperately.^8^ We included ACEIs and ARBs in a single group because the JNC8 panel member report provided differential recommendations for these agents to Black and to non-Black adults. The primary exposure in the model was whether initiation occurred before or after publication of the JNC8 panel member report (January 1, 2011, until December 17, 2013, vs January 1, 2014, until December 31, 2018). The model included adjustment for all covariates listed above. The difference in the change of the proportion initiating an ACEI or an ARB after vs before publication of the JNC8 panel member report among Black, White, and other race/ethnicity beneficiaries was tested by adding interaction terms for race/ethnicity by calendar period to the regression model. The above analyses were repeated to calculate the PRs for initiating antihypertensive medication with a β-blocker vs other antihypertensive classes. A 2-sided *P* < .05 was considered statistically significant. Statistical analyses were conducted using SAS, version 9.3 (SAS Institute Inc).

## Results

In total, 41 340 (65% women; mean [SD] age, 75.7 [7.6] years) Medicare beneficiaries initiated antihypertensive medication and met the inclusion criteria for the present study. Of those included in the analysis, 3303 (8.0%) were Black beneficiaries, 34 943 (84.5%) were White beneficiaries, and 3094 (7.5%) were of other race/ethnicity (Table 1). Overall, 66% of Black beneficiaries initiated antihypertensive medication monotherapy, whereas 78% of White and other race/ethnicity beneficiaries initiated monotherapy (eFigure 2 in the Supplement).

**Table 1.** Characteristics of Medicare Beneficiaries of Black, White, and Other Races/Ethnicities in the 5% Sample Who Initiated Antihypertensive Medication Between 2011 and 2018

Characteristic	Calendar year of antihypertensive medication initiation, No. (%) of each race/ethnicity^a^								*P* value for trend
	2011	2012	2013	2014	2015	2016	2017	2018	
Age, mean (SD), y									
Black	75.6 (7.8)	75.4 (8.1)	75.4 (7.9)	74.6 (7.1)	74.5 (7.8)	74.9 (8.2)	74.7 (7.9)	74.7 (8.0)	.02
White	76.0 (7.5)	76.0 (7.6)	76.0 (7.8)	75.9 (7.7)	75.7 (7.7)	75.9 (7.5)	75.8 (7.5)	75.4 (7.4)	<.001
Other	74.8 (7.1)	76.0 (7.5)	75.2 (7.0)	74.6 (7.6)	74.5 (7.7)	74.1 (7.6)	74.3 (7.4)	74.8 (7.8)	.02
Male sex									
Black	135 (35.3)	157 (36.9)	137 (34.1)	178 (39.8)	157 (40.2)	150 (35.0)	149 (35.4)	161 (39.8)	.43
White	1316 (31.2)	1370 (32.1)	1353 (32.1)	1446 (33.3)	1534 (35.3)	1562 (34.7)	1551 (34.9)	1575 (34.1)	<.001
Other	126 (34.7)	139 (38.1)	137 (39.6)	167 (42.2)	161 (44.4)	174 (40.7)	179 (44.3)	160 (37.3)	.12
Region of residence									
West South Central									
Black	44 (11.5)	50 (11.7)	55 (13.7)	52 (11.6)	52 (13.3)	58 (13.5)	50 (11.9)	57 (14.1)	.36
White	459 (10.9)	469 (11.0)	464 (11.0)	470 (10.8)	446 (10.3)	458 (10.2)	446 (10.0)	480 (10.4)	.06
Other	40 (11.0)	50 (13.7)	61 (17.6)	47 (11.9)	43 (11.8)	52 (12.1)	40 (9.9)	51 (11.9)	.18
Mountain									
Black	Redacted^b^	Redacted^b^	Redacted^b^	Redacted^b^	Redacted^b^	Redacted^b^	Redacted^b^	Redacted^b^	.31
White	224 (5.3)	231 (5.4)	219 (5.2)	230 (5.3)	274 (6.3)	312 (6.9)	295 (6.6)	305 (6.6)	<.001
Other	21 (5.8)	19 (5.2)	16 (4.6)	20 (5.1)	26 (7.2)	35 (8.2)	26 (6.4)	20 (4.7)	.50
East South Central									
Black	47 (12.3)	58 (13.6)	43 (10.7)	53 (11.9)	46 (11.8)	45 (10.5)	45 (10.7)	30 (7.4)	.01
White	343 (8.1)	346 (8.1)	349 (8.3)	330 (7.6)	320 (7.4)	319 (7.1)	296 (6.7)	310 (6.7)	<.001
Other	Redacted^b^	Redacted^b^	Redacted^b^	Redacted^b^	Redacted^b^	Redacted^b^	Redacted^b^	Redacted^b^	.66
Middle Atlantic									
Black	49 (12.8)	47 (11.0)	58 (14.4)	77 (17.2)	58 (14.8)	62 (14.5)	55 (13.1)	66 (16.3)	.15
White	600 (14.2)	598 (14.0)	578 (13.7)	629 (14.5)	630 (14.5)	680 (15.1)	644 (14.5)	662 (14.3)	.30
Other	37 (10.2)	52 (14.2)	43 (12.4)	45 (11.4)	49 (13.5)	53 (12.4)	43 (10.6)	67 (15.6)	.29
South Atlantic									
Black	125 (32.7)	164 (38.5)	151 (37.6)	137 (30.6)	134 (34.3)	135 (31.5)	144 (34.2)	140 (34.6)	.42
White	923 (21.9)	997 (23.4)	957 (22.7)	1001 (23.0)	1005 (23.1)	1039 (23.1)	1063 (23.9)	1094 (23.7)	.05
Other	70 (19.3)	57 (15.6)	50 (14.5)	93 (23.5)	70 (19.3)	76 (17.8)	60 (14.9)	67 (15.6)	.28
West North Central									
Black	Redacted^b^	Redacted^b^	Redacted^b^	Redacted^b^	12 (3.1)	14 (3.3)	12 (2.9)	Redacted^b^	.23
White	392 (9.3)	369 (8.7)	344 (8.2)	315 (7.3)	293 (6.7)	258 (5.7)	278 (6.3)	289 (6.3)	<.001
Other	Redacted^b^	Redacted^b^	Redacted^b^	Redacted^b^	12 (3.3)	Redacted^b^	Redacted^b^	Redacted^b^	.59
East North Central									
Black	74 (19.4)	72 (16.9)	57 (14.2)	85 (19.0)	58 (14.8)	81 (18.9)	71 (16.9)	61 (15.1)	.43
White	638 (15.1)	648 (15.2)	615 (14.6)	643 (14.8)	669 (15.4)	622 (13.8)	629 (14.2)	659 (14.3)	.06
Other	32 (8.8)	20 (5.5)	16 (4.6)	29 (7.3)	34 (9.4)	34 (7.9)	37 (9.2)	34 (7.9)	.18
Pacific									
Black	25 (6.5)	21 (4.9)	15 (3.7)	19 (4.3)	22 (5.6)	19 (4.4)	30 (7.1)	24 (5.9)	.47
White	416 (9.9)	374 (8.8)	437 (10.4)	480 (11.1)	453 (10.4)	554 (12.3)	518 (11.7)	556 (12.0)	<.001
Other	141 (38.8)	144 (39.5)	127 (36.7)	144 (36.4)	117 (32.2)	149 (34.8)	167 (41.3)	164 (38.2)	.94
New England									
Black	Redacted^b^	Redacted^b^	Redacted^b^	Redacted^b^	Redacted^b^	Redacted^b^	Redacted^b^	12 (3.0)	.14
White	217 (5.2)	233 (5.5)	251 (6.0)	245 (5.6)	254 (5.8)	264 (5.9)	270 (6.1)	265 (5.7)	.12
Other	11 (3.0)	12 (3.3)	16 (4.6)	Redacted^b^	Redacted^b^	18 (4.2)	20 (5.0)	Redacted^b^	.80
Cardiologist care									
Black	42 (11.0)	49 (11.5)	46 (11.4)	49 (11.0)	63 (16.1)	53 (12.4)	55 (13.1)	49 (12.1)	.26
White	678 (16.1)	710 (16.6)	711 (16.9)	787 (18.1)	794 (18.3)	791 (17.6)	789 (17.8)	778 (16.8)	.09
Other	60 (16.5)	55 (15.1)	46 (13.3)	57 (14.4)	51 (14.0)	66 (15.4)	57 (14.1)	70 (16.3)	.97
Endocrinologist care									
Black	12 (3.1)	11 (2.6)	Redacted^b^	Redacted^b^	13 (3.3)	Redacted^b^	Redacted^b^	Redacted^b^	.31
White	128 (3.0)	128 (3.0)	113 (2.7)	149 (3.4)	138 (3.2)	192 (4.3)	164 (3.7)	201 (4.4)	<.001
Other	Redacted^b^	Redacted^b^	Redacted^b^	12 (3.0)	12 (3.3)	11 (2.6)	Redacted^b^	15 (3.5)	.24
Nephrologist care									
Black	Redacted^b^	Redacted^b^	Redacted^b^	Redacted^b^	Redacted^b^	Redacted^b^	Redacted^b^	Redacted^b^	.56
White	34 (0.8)	24 (0.6)	24 (0.6)	35 (0.8)	39 (0.9)	28 (0.6)	39 (0.9)	30 (0.6)	.68
Other	Redacted^b^	Redacted^b^	Redacted^b^	Redacted^b^	Redacted^b^	Redacted^b^	Redacted^b^	Redacted^b^	.47
Low income subsidy/Medicare-Medicaid eligible									
Black	234 (61.3)	253 (59.4)	229 (57.0)	196 (43.8)	171 (43.7)	185 (43.1)	182 (43.2)	161 (39.8)	<.001
White	860 (20.4)	813 (19.1)	758 (18.0)	673 (15.5)	607 (14.0)	688 (15.3)	669 (15.1)	598 (12.9)	<.001
Other	250 (68.9)	247 (67.7)	221 (63.9)	233 (58.8)	205 (56.5)	201 (47.0)	207 (51.2)	210 (49.0)	<.001
Area-level median income<$25 000									
Black	28 (7.3)	28 (6.6)	28 (7.0)	22 (4.9)	26 (6.6)	30 (7.0)	21 (5.0)	14 (3.5)	.03
White	24 (0.6)	33 (0.8)	22 (0.5)	22 (0.5)	30 (0.7)	18 (0.4)	18 (0.4)	11 (0.2)	<.001
Other	Redacted^b^	Redacted^b^	Redacted^b^	Redacted^b^	Redacted^b^	Redacted^b^	Redacted^b^	Redacted^b^	.10

^a^ Number of Black, White, and other race/ethnicity beneficiaries in each calendar year. For 2011: Black = 382, White = 4212, other = 363; for 2012: Black = 426, White = 4265, other = 365; for 2013: Black = 402, White = 4214, other = 346; for 2014: Black = 447, White = 4343, other = 396; for 2015: Black = 391, White = 4344, other = 363; for 2016: Black = 429, White = 4506, other = 428; for 2017: Black = 421, White = 4439, other = 404; for 2018: Black = 405, White = 4620, other = 429; overall: Black = 3303, White = 34 943, other = 3094.

^b^ Owing to small numbers.

### Initiation of Antihypertensive Medication Monotherapy

**Figure 1.**
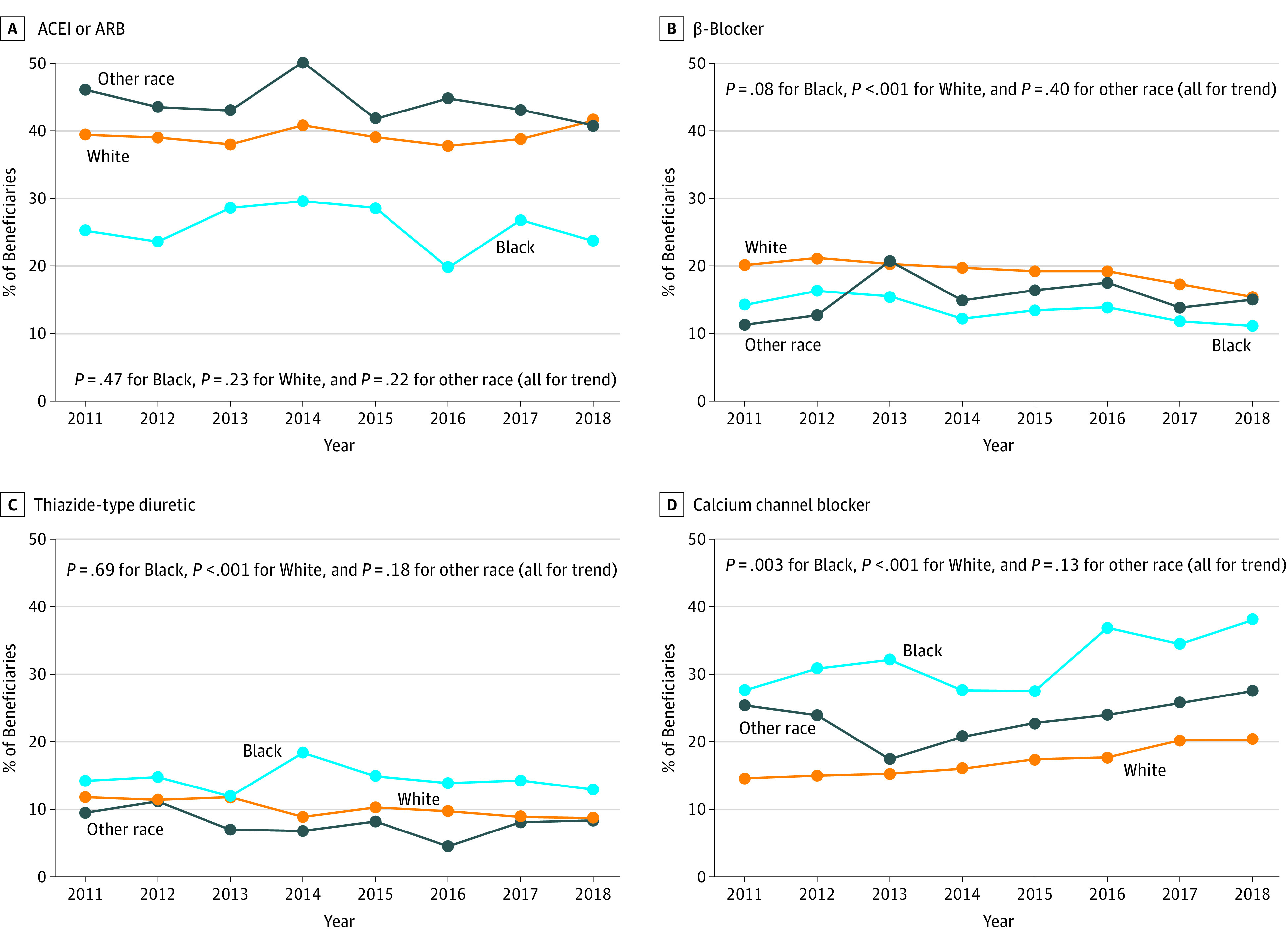
Proportion of Medicare Beneficiaries of Black, White, and Other Races/Ethnicities Initiating Monotherapy With an Angiotensin-Converting Enzyme Inhibitor (ACEI) or Angiotensin Receptor Blocker (ARB), β-Blocker, Thiazide-Type Diuretic, or Calcium Channel Blocker, by Calendar Year

The proportion of Black beneficiaries initiating antihypertensive monotherapy who did so with an ACEI or ARB was 25.2% in 2011 and 23.7% in 2018 (*P* = .47 for trend; Figure 1; eTable 4 in the Supplement). Among both Black beneficiaries and White beneficiaries, the proportion initiating monotherapy with an ACEI decreased, and the proportion initiating with an ARB increased. The proportion of beneficiaries initiating monotherapy who did so with a β-blocker was 20.1% in 2011 and 15.4% in 2018 among White beneficiaries (*P* < .001 for trend), 14.2% in 2011 and 11.1% in 2018 among Black beneficiaries (*P* = .08 for trend), and 11.3% in 2011 and 15.0% in 2018 among beneficiaries of other race/ethnicity (*P* = .40 for trend). There was no evidence of trends in the proportion of Black beneficiaries or beneficiaries of other race/ethnicity initiating monotherapy with a thiazide-type diuretic. The proportion of White beneficiaries initiating monotherapy with a thiazide-type diuretic decreased. The proportion of beneficiaries initiating CCB monotherapy increased from 27.6% in 2011 to 38.0% in 2018 among Black beneficiaries (*P* = .003 for trend) and from 14.6% in 2011 to 20.3% in 2018 among White beneficiaries (*P* < .001 for trend). There was no evidence of a change in the proportion of beneficiaries of other race/ethnicity who initiated CCB monotherapy.

### Initiation of Antihypertensive Medication Combination Therapy

**Figure 2.**
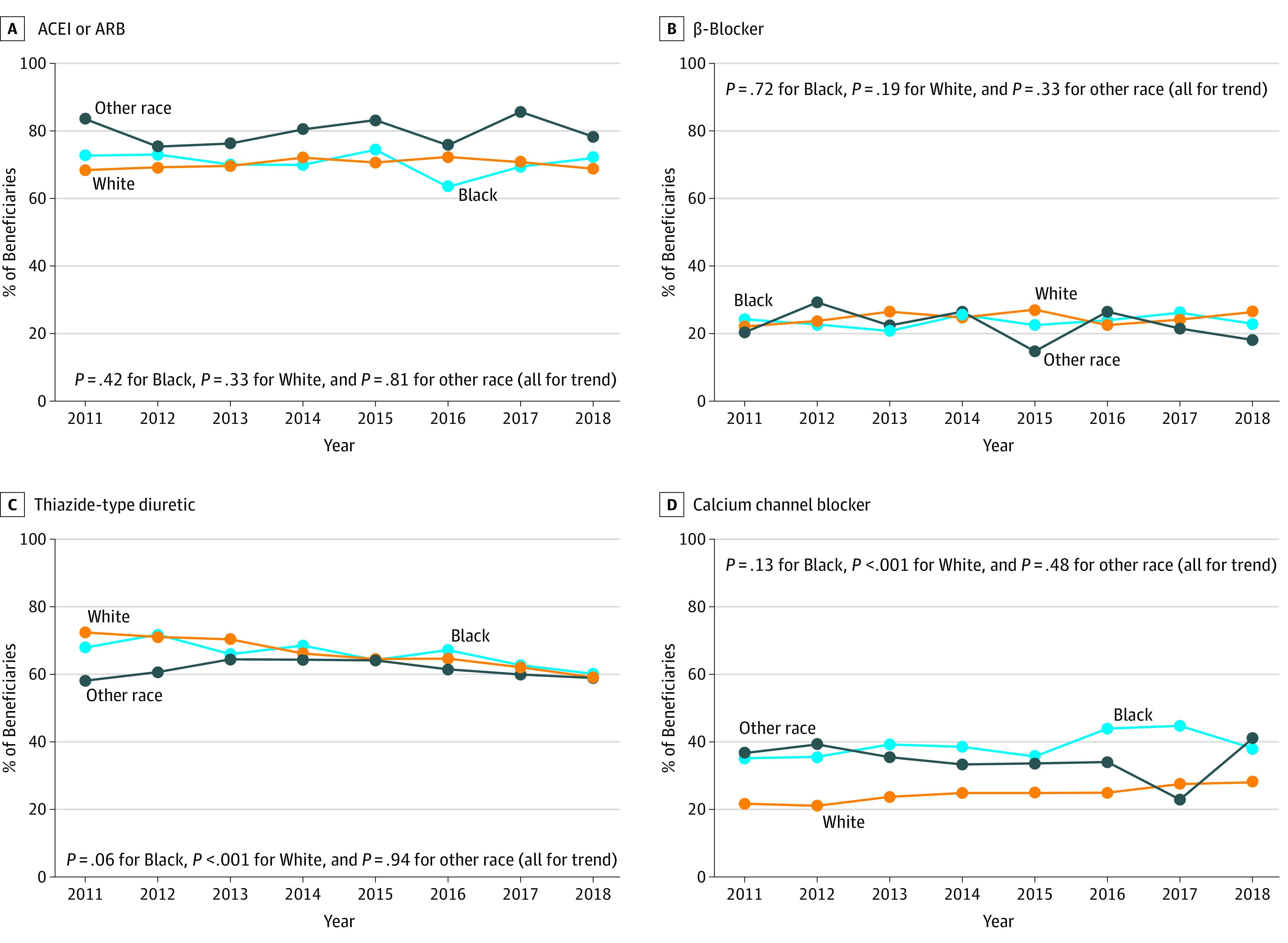
Proportion of Medicare Beneficiaries of Black, White, and Other Races/Ethnicities Initiating Combination Therapy With an Angiotensin-Converting-Enzyme Inhibitor (ACEI) or Angiotensin Receptor Blocker (ARB), β-Blocker, Thiazide-Type Diuretic, or Calcium Channel Blocker, by Calendar Year

There was no evidence for trends in the proportion of Black beneficiaries or beneficiaries of other race/ethnicity initiating antihypertensive combination therapy who did so with an ACEI or ARB, β-blocker, thiazide-type diuretic, or CCB from 2011 to 2018 (Figure 2; eTable 5 in the Supplement). The proportion of White beneficiaries initiating combination therapy who did so with an ARB or CCB increased, whereas the proportion initiating treatment with a thiazide-type diuretic decreased. The most common dual antihypertensive combination therapy filled by beneficiaries in each race/ethnicity group in 2018 was a thiazide-type diuretic with either an ACEI or an ARB (eTable 6 in the Supplement).

### Factors Associated With Initiation of an ACEI or an ARB

There was no evidence of a change in the proportion of beneficiaries initiating monotherapy who did so with an ACEI or ARB after vs before publication of the JNC8 panel member report (PR, 1.00; 95% CI, 0.97-1.03) (Table 2). Black beneficiaries were less likely than White beneficiaries to initiate monotherapy with an ACEI or ARB (PR, 0.68; 95% CI, 0.60-0.77), but there was no evidence of a change in the proportion of Black vs White beneficiaries initiating ACEI or ARB monotherapy after vs before publication of JNC8 panel member report (PR, 0.96; 95% CI, 0.83-1.12; *P* = .60 for interaction). There was no evidence of a change in the proportion of beneficiaries initiating combination therapy who did so with an ACEI or ARB after vs before publication of the JNC8 panel member report, overall or across race/ethnicity groups.

**Table 2.** Initiation of Antihypertensive Medication With a Renin-Angiotensin-Aldosterone System Inhibitor (ie, ACEI or ARB) Among Those Initiating Antihypertensive Monotherapy and, Separately, Initiating Combination Therapy

Variable	Prevalence ratio (95% CI)^a^^,^^b^	
	Beneficiaries initiating monotherapy (n = 31 909)	Beneficiaries initiating combination therapy (n = 9431)
Initiated antihypertensive medication after vs before publication of JNC8 panel member report^c^	1.00 (0.97-1.03)	1.02 (0.99-1.05)
Race/ethnicity		
White	1 [Reference]	1 [Reference]
Black	0.68 (0.60-0.77)	1.02 (0.95-1.09)
Other	0.94 (0.81-1.09)	1.04 (0.91-1.19)
Age, y		
65-69	1 [Reference]	1 [Reference]
70-74	0.97 (0.94-1.01)	0.97 (0.93-1.00)
75-79	0.92 (0.89-0.96)	0.97 (0.93-1.00)
80-84	0.84 (0.80-0.88)	0.92 (0.88-0.96)
≥85	0.67 (0.64-0.71)	0.88 (0.84-0.92)
Male sex	1.17 (1.13-1.20)	1.11 (1.08-1.14)
Geographical region of residence		
West South Central	1 [Reference]	1 [Reference]
Mountain	1.17 (1.10-1.25)	0.94 (0.88-1.00)
East South Central	0.94 (0.87-1.01)	0.93 (0.88-0.99)
Middle Atlantic	0.92 (0.87-0.98)	0.92 (0.88-0.97)
South Atlantic	1.05 (1.00-1.11)	0.91 (0.88-0.95)
West North Central	1.00 (0.93-1.07)	0.89 (0.84-0.95)
East North Central	0.97 (0.92-1.03)	0.92 (0.88-0.96)
Pacific	1.15 (1.09-1.22)	0.93 (0.89-0.98)
New England	0.94 (0.87-1.01)	0.89 (0.83-0.96)
Cardiologist care	0.87 (0.83-0.90)	1.04 (1.00-1.07)
Endocrinologist care	0.92 (0.85-1.00)	0.93 (0.85-1.02)
Nephrologist care	0.69 (0.56-0.85)	0.92 (0.76-1.10)
Medicare-Medicaid eligible/low income subsidy	0.88 (0.85-0.91)	1.02 (0.99-1.06)
Area-level median income, $		
<25 000	1 [Reference]	1 [Reference]
25 000-49 999	0.89 (0.76-1.04)	0.94 (0.85-1.05)
50 000-74 999	0.88 (0.76-1.03)	0.96 (0.87-1.06)
≥75 000	0.91 (0.78-1.07)	0.96 (0.86-1.07)

Abbreviations: ACEI, angiotensin-converting enzyme inhibitor; ARB, angiotensin receptor blocker; JNC8, Eighth Joint National Committee on Prevention, Detection, Evaluation, and Treatment of High Blood Pressure.

^a^ Prevalence ratios adjusted for antihypertensive medication initiation period, race/ethnicity, antihypertensive medication initiation period × race/ethnicity, age, sex, geographic region of residence, cardiologist care, endocrinologist care, nephrologist care, dual eligibility for Medicare-Medicaid/low income subsidy, and residence area–level median income.

^b^ *P* values for interaction for initiating antihypertensive medication after vs before December 18, 2013: by Black vs White race/ethnicity for monotherapy, *P* = .60; by other vs White race/ethnicity for monotherapy, *P* = .35; by Black vs White race/ethnicity for combination therapy, *P* = .20; by other vs White race/ethnicity for combination therapy, *P* = .76.

^c^ The JNC8 panel member report was published online December 18, 2013.

### Factors Associated With Initiation of a β-Blocker

The proportion of beneficiaries initiating antihypertensive monotherapy who did so with a β-blocker decreased from before to after publication of the JNC8 panel member report (PR, 0.89; 95% CI, 0.84-0.93) (Table 3). Black beneficiaries were less likely than White beneficiaries to initiate monotherapy with a β-blocker (PR, 0.83; 95% CI, 0.70-0.99). Among beneficiaries who initiated combination therapy, a higher proportion of other race/ethnicity compared with White beneficiaries had a regimen that included a β-blocker (PR, 1.50; 95% CI, 1.09-2.06).

**Table 3.** Initiation of Antihypertensive Medication With a β-Blocker Among Those Initiating Antihypertensive Monotherapy and, Separately, Initiating Combination Therapy

Variable	Prevalence ratio (95% CI)^a^^,^^b^	
	Beneficiaries initiating monotherapy (n = 31 909)	Beneficiaries initiating combination therapy (n = 9431)
Initiated antihypertensive medication after vs before publication of JNC8 panel member report^c^	0.89 (0.84 0.93)	1.05 (0.97 1.14)
Race/ethnicity		
White	1 [Reference]	1 [Reference]
Black	0.83 (0.70-0.99)	0.87 (0.73-1.05)
Other	0.90 (0.70-1.17)	1.50 (1.09-2.06)
Age, y		
65-69	1 [Reference]	1 [Reference]
70-74	1.02 (0.96-1.09)	1.04 (0.94-1.14)
75-79	0.99 (0.92-1.06)	0.98 (0.88-1.10)
80-84	1.06 (0.98-1.14)	1.24 (1.10-1.39)
≥85	0.93 (0.86-1.01)	1.30 (1.17-1.46)
Male	0.89 (0.85-0.94)	0.99 (0.92-1.07)
Geographical region of residence		
West South Central	1 [Reference]	1 [Reference]
Mountain	0.88 (0.77-1.00)	1.08 (0.89-1.30)
East South Central	1.03 (0.92-1.15)	1.00 (0.84-1.19)
Middle Atlantic	1.09 (1.00-1.20)	1.20 (1.04-1.39)
South Atlantic	0.94 (0.86-1.02)	1.11 (0.97-1.26)
West North Central	1.12 (1.00-1.25)	1.28 (1.09-1.51)
East North Central	1.08 (0.99-1.19)	1.24 (1.08-1.42)
Pacific	0.91 (0.83-1.01)	0.98 (0.83-1.15)
New England	1.08 (0.96-1.22)	1.37 (1.13-1.66)
Cardiologist care	1.60 (1.52-1.68)	1.50 (1.37-1.63)
Endocrinologist care	1.09 (0.97-1.22)	0.99 (0.79-1.24)
Nephrologist care	0.88 (0.66-1.16)	0.85 (0.52-1.36)
Medicare-Medicaid eligible/low income subsidy	0.92 (0.87-0.98)	1.26 (1.16-1.37)
Area-level median income, $		
<25 000	1 [Reference]	1 [Reference]
25 000-49 999	1.74 (1.21-2.51)	0.93 (0.70-1.22)
50 000-74 999	1.75 (1.21-2.52)	0.84 (0.63-1.11)
≥75 000	1.67 (1.15-2.41)	0.85 (0.64-1.13)

Abbreviations: JNC8, Eighth Joint National Committee on Prevention, Detection, Evaluation, and Treatment of High Blood Pressure.

^a^ Prevalence ratios adjusted for antihypertensive medication initiation period, race/ethnicity, antihypertensive medication initiation period × race/ethnicity, age, sex, geographic region of residence, cardiologist care, endocrinologist care, nephrologist care, dual eligibility for Medicare-Medicaid/low income subsidy, and residence area-level median income.

^b^ *P* values for interaction for initiating antihypertensive medication after vs before December 18, 2013: by Black vs White race/ethnicity for monotherapy, *P* = .30; by other vs White race/ethnicity for monotherapy, *P* = .48; by Black vs White race/ethnicity for combination therapy, *P* = .52; by other vs White race/ethnicity for combination therapy, *P* = .06.

^c^ The JNC8 panel member report was published online December 18, 2013.

## Discussion

The current study suggests that there was no change in the proportion of Black Medicare beneficiaries initiating an ACEI or ARB as monotherapy in the period after compared with before publication of the JNC8 panel member report. Initiation of CCB monotherapy increased from 2011 to 2018 among both Black beneficiaries and White beneficiaries. However, use of β-blockers as initial monotherapy was high and decreased only modestly. Many older US adults who initiated antihypertensive medication did so with non–guideline-recommended classes of medication.

Although Black Medicare beneficiaries initiating antihypertensive monotherapy were less likely than White beneficiaries to do so with an ACEI or ARB, a high proportion initiated treatment with an ACEI or ARB, and this did not change from before to after publication of the JNC8 panel member report. There are decades of evidence suggesting racial differences in BP-lowering and CVD risk associated with renin-angiotensin-aldosterone system inhibitors.^3,9,10^ However, studies examining trends in antihypertensive medication use among US adults since the 2000s have not reported decreases in the use of these agents among Black adults.^11,12^ In the current study, more than 20% of Black Medicare beneficiaries without compelling indications for ACEI or ARB monotherapy initiated these classes of antihypertensive medication in 2018.

Although there were no changes from 2011 to 2018 in initiation of monotherapy with either an ACEI or ARB among any race/ethnicity group, there were changes in initiation of these agents when considered separately. Among Black beneficiaries, and separately, White beneficiaries initiating monotherapy, the proportion initiating treatment with an ACEI decreased, whereas the proportion initiating with ARB increased. The decrease in ACEI use and increase in ARB use may be due to evidence of their similar efficacy^13,14^ and higher risk of adverse effects associated with ACEIs,^13^ especially among Black individuals.^15,16^

The proportion of beneficiaries initiating monotherapy who did so with a β-blocker decreased following publication of the JNC8 panel member report. Despite this decrease, in 2018 a high proportion of beneficiaries initiating monotherapy did so with a β-blocker. The JNC8 panel member report and the 2017 ACC/AHA hypertension guideline recommended that β-blockers not be used as initial therapy for hypertension in adults without CHD or without heart failure with reduced ejection fraction.^1,6^ This recommendation was based on evidence of higher risks of CVD^5,17,18^ and CVD mortality^17^ in patients with hypertension treated with a β-blocker compared with other antihypertensive medication classes. In addition, a meta-analysis of randomized clinical trials found that among adults 60 years of age or older, the risk for stroke was higher for β-blockers compared with other classes of antihypertensive medication.^19^ The continued prescribing for β-blockers for initial antihypertensive monotherapy represents a missed opportunity to decrease CVD risk among adults with hypertension.

Although initiation of monotherapy with non–guideline-recommended medications remained high at the end of the study period, there was evidence that some prescribing practices may have changed from before to after publication of the JNC8 panel member report. In the present analysis, fills for CCBs among Black beneficiaries initiating monotherapy increased during the years studied. This pattern has been present elsewhere.^20^ A retrospective study examining changes in initial antihypertensive monotherapy across ethnic groups after publication of the 2006 National Institute for Health and Care Excellence hypertension guideline found that prescribing of CCBs increased among Black adults in the UK.^20^ These findings suggest the need for research focused on effective strategies for implementing guideline-recommended prescribing practices.

Findings from the present study suggest that the race/ethnicity-specific recommendation for initial antihypertensive medication included in the JNC8 panel member report was not widely adopted. Lack of guideline adoption is a well-known,^21,22^ multifaceted issue involving factors associated with patients, clinicians, health care institutions, and the guidelines themselves.^23,24^ Adoption of the race/ethnicity-specific recommendation from the JNC8 panel member report may have been hindered by the controversies that surrounded its publication,^25^ including a dissenting opinion by some of the panel members.^26^ In addition, the race/ethnicity-specific recommendation was only of moderate strength and certainty (Grade B, moderate recommendation).^1^ The 2017 ACC/AHA hypertension guideline also includes the race/ethnicity-specific recommendation for initial antihypertensive medication classes.^6^ However, the recommendation is stronger (Class I; level of evidence, B-R). Sufficient data were not available in the current study to assess whether the race/ethnicity-specific recommendation included in the 2017 ACC/AHA hypertension guideline affected prescribing patterns, but this should be investigated in future studies.

### Strengths and Limitations

The present study has several strengths. We used data from a 5% random sample of Medicare beneficiaries. The majority of US adults 65 years of age or older have health insurance through the Medicare program, allowing for high generalizability to older US adults. The use of Medicare claims enabled the identification of the initial antihypertensive medication regimens filled by adults without compelling indications. This study also has limitations. The modest sample sizes of Black beneficiaries and beneficiaries of other race/ethnicity may have limited the ability to detect statistically significant changes in classes of antihypertensive medications initiated over time. We did not have data on BP levels; thus, we could not stratify results by BP levels at the time of medication initiation or assess whether beneficiaries achieved BP control with regimen that they initiated. We assumed the medication claims filled reflected prescribing decisions made by the beneficiaries’ clinicians, but it is possible that patients themselves requested non–guideline-recommended therapies. We did not exclude beneficiaries with cognitive impairment from the analysis, an outcome domain that ACEIs and ARBs may affect beneficially.^27^ We used Medicare claims to identify pharmacy fills and exclude beneficiaries with a history of compelling indications. Incomplete exclusion of beneficiaries with compelling indications could have biased the results because the general recommendation for initial antihypertensive medication therapy included in the JNC8 panel member report would not apply to those with compelling indications.

## Conclusions

From 2011 to 2018, the proportion of Black Medicare beneficiaries initiating monotherapy who did so with an ACEI decreased, and the proportion initiating with an ARB increased. However, there was no evidence that the proportion of Black Medicare beneficiaries initiating monotherapy with either an ACEI or ARB changed from before to after publication of the JNC8 panel member report. Furthermore, despite a modest decrease in the period following publication of the JNC8 panel member report, a substantial proportion of Medicare beneficiaries who initiated antihypertensive monotherapy did so with a β-blocker. Achieving population-wide BP goals and maximizing the CVD risk decrease associated with antihypertensive medication will require efforts to increase guideline-recommended choices among US adults initiating antihypertensive medication.

## Notes

Supplement. **eTable 1.** Antihypertensive Medications Used to Define Medication Class Initiation **eTable 2.** Compelling Indications for Individual Antihypertensive Medication Classes as Recommended by JNC7 **eTable 3.** Definitions for Beneficiary Characteristics and Compelling Indications for Specific Antihypertensive Medication Classes **eTable 4.** Number and Percentage of Medicare Beneficiaries of Black, White, and Other Races/Ethnicities Initiating Each Class of Antihypertensive Medication, by Calendar Year Among Those Initiating Antihypertensive Medication Monotherapy **eTable 5.** Number and Percentage of Medicare Beneficiaries of Black, White, and Other Races/Ethnicities Initiating Each Class of Antihypertensive Medication, by Calendar Year Among Those Initiating Antihypertensive Combination Therapy **eTable 6.** Number and Percentage of Medicare Beneficiaries of Black, White, and Other Races/Ethnicities Initiating the Most Common Combinations of Dual and Triple Therapies, by Calendar Year Among Those Initiating Two or More Classes of Antihypertensive Medication **eFigure 1.** Flowchart for Determining the Eligible Study Population **eFigure 2.** Proportion of Medicare Beneficiaries of Black, White, and Other Races/Ethnicities Initiating One, Two, Three, or Four or More Antihypertensive Medication Classes by Calendar Year **eReferences.**

## References

[1] James PA, Oparil S, Carter BL, 2014 Evidence-based guideline for the management of high blood pressure in adults: report from the panel members appointed to the Eighth Joint National Committee (JNC 8). JAMA. 2014;311(5):507-520. doi:10.1001/jama.2013.284427 24352797

[2] Chobanian AV, Bakris GL, Black HR, ; National Heart, Lung, and Blood Institute Joint National Committee on Prevention, Detection, Evaluation, and Treatment of High Blood Pressure; National High Blood Pressure Education Program Coordinating Committee The Seventh Report of the Joint National Committee on Prevention, Detection, Evaluation, and Treatment of High Blood Pressure: the JNC 7 report. JAMA. 2003;289(19):2560-2572. doi:10.1001/jama.289.19.2560 12748199

[3] Officers A; ALLHAT Officers and Coordinators for the ALLHAT Collaborative Research Group. The Antihypertensive and Lipid-Lowering Treatment to Prevent Heart Attack Trial Major outcomes in high-risk hypertensive patients randomized to angiotensin-converting enzyme inhibitor or calcium channel blocker vs diuretic: the Antihypertensive and Lipid-Lowering Treatment to Prevent Heart Attack Trial (ALLHAT). JAMA. 2002;288(23):2981-2997. doi:10.1001/jama.288.23.2981 12479763

[4] Leenen FHH, Nwachuku CE, Black HR, ; Antihypertensive and Lipid-Lowering Treatment to Prevent Heart Attack Trial Collaborative Research Group Clinical events in high-risk hypertensive patients randomly assigned to calcium channel blocker versus angiotensin-converting enzyme inhibitor in the antihypertensive and lipid-lowering treatment to prevent heart attack trial. Hypertension. 2006;48(3):374-384. doi:10.1161/01.HYP.0000231662.77359.de 16864749

[5] Dahlöf B, Devereux RB, Kjeldsen SE, ; LIFE Study Group Cardiovascular morbidity and mortality in the Losartan Intervention for Endpoint Reduction in Hypertension Study (LIFE): a randomised trial against atenolol. Lancet. 2002;359(9311):995-1003. doi:10.1016/S0140-6736(02)08089-3 11937178

[6] Whelton PK, Carey RM, Aronow WS, 2017 ACC/AHA/AAPA/ABC/ACPM/AGS/APhA/ASH/ASPC/NMA/PCNA guideline for the prevention, detection, evaluation, and management of high blood pressure in adults: a report of the American College of Cardiology/American Heart Association Task Force on Clinical Practice Guidelines. J Am Coll Cardiol. 2018;71(19):e127-e248. doi:10.1016/j.jacc.2017.11.006 29146535

[7] United States Census Bureau. 2010 Census regions and divisions of the United States. Updated August 20, 2018. Accessed August 20, 2020. https://www.census.gov/geographies/reference-maps/2010/geo/2010-census-regions-and-divisions-of-the-united-states.html

[8] Zou G A modified Poisson regression approach to prospective studies with binary data. Am J Epidemiol. 2004;159(7):702-706. doi:10.1093/aje/kwh090 15033648

[9] Saunders E, Weir MR, Kong BW, A comparison of the efficacy and safety of a β-blocker, a calcium channel blocker, and a converting enzyme inhibitor in hypertensive blacks. Arch Intern Med. 1990;150(8):1707-1713. doi:10.1001/archinte.1990.00040031707020 2200382

[10] Cushman WC, Reda DJ, Perry HM, Williams D, Abdellatif M, Materson BJ; Department of Veterans Affairs Cooperative Study Group on Antihypertensive Agents Regional and racial differences in response to antihypertensive medication use in a randomized controlled trial of men with hypertension in the United States. Arch Intern Med. 2000;160(6):825-831. doi:10.1001/archinte.160.6.825 10737282

[11] Gu Q, Burt VL, Dillon CF, Yoon S Trends in antihypertensive medication use and blood pressure control among United States adults with hypertension: the National Health And Nutrition Examination Survey, 2001 to 2010. Circulation. 2012;126(17):2105-2114. doi:10.1161/CIRCULATIONAHA.112.096156 23091084

[12] Derington CG, King JB, Herrick JS, Trends in antihypertensive medication monotherapy and combination use among US adults, National Health and Nutrition Examination Survey 2005-2016. Hypertension. 2020;75(4):973-981. doi:10.1161/HYPERTENSIONAHA.119.14360 32148129 PMC7398637

[13] Matchar DB, McCrory DC, Orlando LA, Systematic review: comparative effectiveness of angiotensin-converting enzyme inhibitors and angiotensin II receptor blockers for treating essential hypertension. Ann Intern Med. 2008;148(1):16-29. doi:10.7326/0003-4819-148-1-200801010-00189 17984484

[14] Messerli FH, Bangalore S, Bavishi C, Rimoldi SF Angiotensin-converting enzyme inhibitors in hypertension: to use or not to use? J Am Coll Cardiol. 2018;71(13):1474-1482. doi:10.1016/j.jacc.2018.01.058 29598869

[15] Brown NJ, Ray WA, Snowden M, Griffin MR Black Americans have an increased rate of angiotensin converting enzyme inhibitor-associated angioedema. Clin Pharmacol Ther. 1996;60(1):8-13. doi:10.1016/S0009-9236(96)90161-7 8689816

[16] Kostis JB, Kim HJ, Rusnak J, Incidence and characteristics of angioedema associated with enalapril. Arch Intern Med. 2005;165(14):1637-1642. doi:10.1001/archinte.165.14.1637 16043683

[17] Reboussin DM, Allen NB, Griswold ME, Systematic Review for the 2017 ACC/AHA/AAPA/ABC/ACPM/AGS/APhA/ASH/ASPC/NMA/PCNA Guideline for the Prevention, Detection, Evaluation, and Management of High Blood Pressure in Adults: a report of the American College of Cardiology/American Heart Association Task Force on Clinical Practice Guidelines. Hypertension. 2018;71(6):e116-e135. doi:10.1161/HYP.0000000000000067 29133355

[18] Psaty BM, Lumley T, Furberg CD, Health outcomes associated with various antihypertensive therapies used as first-line agents: a network meta-analysis. JAMA. 2003;289(19):2534-2544. doi:10.1001/jama.289.19.2534 12759325

[19] Khan N, McAlister FA Re-examining the efficacy of β-blockers for the treatment of hypertension: a meta-analysis. CMAJ. 2006;174(12):1737-1742. doi:10.1503/cmaj.060110 16754904 PMC1471831

[20] Barrera L, Leaper C, Pape UJ, Majeed A, Blangiardo M, Millett C Impact of ethnic-specific guidelines for anti-hypertensive prescribing in primary care in England: a longitudinal study. BMC Health Serv Res. 2014;14:87. doi:10.1186/1472-6963-14-87 24568655 PMC3943578

[21] Siegel D, Lopez J Trends in antihypertensive drug use in the United States: do the JNC V recommendations affect prescribing? JAMA. 1997;278(21):1745-1748. doi:10.1001/jama.1997.03550210043036 9388150

[22] Clause SL, Hamilton RA Medicaid prescriber compliance with Joint National Committee VI Hypertension Treatment Guidelines. Ann Pharmacother. 2002;36(10):1505-1511. doi:10.1345/aph.1A451 12243597

[23] Cabana MD, Rand CS, Powe NR, Why don’t physicians follow clinical practice guidelines? a framework for improvement. JAMA. 1999;282(15):1458-1465. doi:10.1001/jama.282.15.1458 10535437

[24] Fischer F, Lange K, Klose K, Greiner W, Kraemer A Barriers and strategies in guideline implementation—a scoping review. Healthcare (Basel). 2016;4(3):36. doi:10.3390/healthcare4030036 27417624 PMC5041037

[25] O’Brien E End of the Joint National Committee heritage? Hypertension. 2014;63(5):904-906. doi:10.1161/HYPERTENSIONAHA.113.03097 24491389

[26] Wright JT Jr, Fine LJ, Lackland DT, Ogedegbe G, Dennison Himmelfarb CR Evidence supporting a systolic blood pressure goal of less than 150 mm Hg in patients aged 60 years or older: the minority view. Ann Intern Med. 2014;160(7):499-503. doi:10.7326/M13-2981 24424788

[27] Levi Marpillat N, Macquin-Mavier I, Tropeano AI, Bachoud-Levi AC, Maison P Antihypertensive classes, cognitive decline and incidence of dementia: a network meta-analysis. J Hypertens. 2013;31(6):1073-1082. doi:10.1097/HJH.0b013e3283603f53 23552124

