# Supplementary material for: Association of Race/Ethnicity-Specific Changes in Antihypertensive Medication Classes Initiated Among Medicare Beneficiaries With the Eighth Joint National Committee Panel Member Report
Source: JAMA Netw Open. 2020 Nov 18;3(11):e2025127. doi: 10.1001/jamanetworkopen.2020.25127 (PMC7675104; doi:10.1001/jamanetworkopen.2020.25127)
Supplement: Supplement. — eTable 1. Antihypertensive Medications Used to Define Medication Class Initiation eTable 2. Compelling Indications for Individual Antihypertensive Medication Classes as Recommended by JNC7 eTable 3. Definitions for Beneficiary Characteristics and Compelling Indications for Specific Antihypertensive Medication Classes eTable 4. Number and Percentage of Medicare Beneficiaries of Black, White, and Other Races/Ethnicities Initiating Each Class of Antihypertensive Medication, by Calendar Year Among Those Initiating Antihypertensive Medication Monotherapy eTable 5. Number and Percentage of Medicare Beneficiaries of Black, White, and Other Races/Ethnicities Initiating Each Class of Antihypertensive Medication, by Calendar Year Among Those Initiating Antihypertensive Combination Therapy eTable 6. Number and Percentage of Medicare Beneficiaries of Black, White, and Other Races/Ethnicities Initiating the Most Common Combinations of Dual and Triple Therapies, by Calendar Year Among Those Initiating Two or More Classes of Antihypertensive Medication eFigure 1. Flowchart for Determining the Eligible Study Population eFigure 2. Proportion of Medicare Beneficiaries of Black, White, and Other Races/Ethnicities Initiating One, Two, Three, or Four or More Antihypertensive Medication Classes by Calendar Year eReferences. [file jamanetwopen-e2025127-s001.pdf]

Supplementary Online Content

Colvin CL, King JB, Oparil S, et al. Association of race/ethnicity-specific changes in antihypertensive medication classes initiated among Medicare beneficiaries with the Eighth Joint National Committee panel member report. *JAMA Netw Open*. 2020;3(11):e2025127. doi:10.1001/jamanetworkopen.2020.25127

eTable 1. Antihypertensive Medications Used to Define Medication Class Initiation

eTable 2. Compelling Indications for Individual Antihypertensive Medication Classes as Recommended by JNC7

eTable 3. Definitions for Beneficiary Characteristics and Compelling Indications for Specific Antihypertensive Medication Classes

eTable 4. Number and Percentage of Medicare Beneficiaries of Black, White, and Other Races/Ethnicities Initiating Each Class of Antihypertensive Medication, by Calendar Year Among Those Initiating Antihypertensive Medication Monotherapy

eTable 5. Number and Percentage of Medicare Beneficiaries of Black, White, and Other Races/Ethnicities Initiating Each Class of Antihypertensive Medication, by Calendar Year Among Those Initiating Antihypertensive Combination Therapy

eTable 6. Number and Percentage of Medicare Beneficiaries of Black, White, and Other Races/Ethnicities Initiating the Most Common Combinations of Dual and Triple Therapies, by Calendar Year Among Those Initiating Two or More Classes of Antihypertensive Medication

eFigure 1. Flowchart for Determining the Eligible Study Population

eFigure 2. Proportion of Medicare Beneficiaries of Black, White, and Other Races/Ethnicities Initiating One, Two, Three, or Four or More Antihypertensive Medication Classes by Calendar Year

eReferences.

This supplementary material has been provided by the authors to give readers additional information about their work.

© 2020 Colvin CL et al. *JAMA Network Open*.

eTable 1. Antihypertensive Medications Used to Define Medication Class Initiation

Antihypertensive Medication Class	Drug Name
Angiotensin-converting enzyme inhibitors (ACEI)	Benazepril<br>Captopril<br>Enalapril<br>Fosinopril<br>Lisinopril<br>Moexipril<br>Perindopril<br>Quinapril<br>Ramipril<br>Trandolapril
Angiotensin receptor blockers (ARB)	Azilsartan<br>Candesartan<br>Eprosartan<br>Irbesartan<br>Losartan<br>Olmesartan<br>Telmisartan<br>Valsartan
Thiazide/thiazide-type diuretics	Chlorothiazide<br>Chlorthalidone<br>Hydrochlorothiazide (HCTZ)<br>Indapamide<br>Metolazone
Potassium-sparing diuretics	Amiloride<br>Triamterene
Loop Diuretics	Bumetanide<br>Furosemide<br>Torsemide
Aldosterone receptor antagonists	Eplerenone<br>Spironolactone
Beta-blockers	Atenolol<br>Betaxolol<br>Bisoprolol<br>Metoprolol<br>Nebivolol<br>Nadolol<br>Propanolol<br>Acebutolol<br>Carteolol<br>Penbutolol<br>Pindolol<br>Carvedilol<br>Labetalol
Calcium channel blockers	Amlodipine<br>Felodipine<br>Isradipine<br>Nicardipine<br>Nifedipine<br>Nisoldipine<br>Diltiazem

© 2020 Colvin CL et al. *JAMA Network Open*.

Antihypertensive Medication Class		Drug Name
		Verapamil
Other	Alpha blockers	Doxazosin<br>Prazosin<br>Terazosin
	Central acting agents	Clonidine<br>Methyldopa<br>Guanfacine
	Direct vasodilators	Hydralazine<br>Minoxidil
	Renin inhibitor	Aliskiren

© 2020 Colvin CL et al. *JAMA Network Open*.

eTable 2. Compelling Indications for Individual Antihypertensive Medication Classes as Recommended by JNC7[1]

Compelling Indication	Initial Antihypertensive Medical Class Recommended
Heart failure	Thiazide-type diuretic, beta-blocker, ACEI, ARB, aldosterone antagonist
Post-myocardial infarction	Beta-blocker, ACEI, ARB, aldosterone antagonist
Diabetes	Thiazide-type diuretic, ACEI, ARB, calcium channel blocker
Chronic Kidney Disease	ACEI, ARB
History of stroke	Thiazide-type diuretic, ACEI

ACEI: Angiotensin converting enzyme inhibitor, ARB: Angiotensin receptor blocker.; JNC7: The Seventh Report of the Joint National Committee on Prevention, Detection, Evaluation, and Treatment of High Blood Pressure.

© 2020 Colvin CL et al. *JAMA Network Open*.

eTable 3. Definitions for Beneficiary Characteristics and Compelling Indications for Specific Antihypertensive Medication Classes

Variables	Definition
Beneficiary Characteristics	
Age	Calculated on the last day of the look-back period using birthdates from the Medicare beneficiary summary files.
Sex, race/ethnicity	As defined in Medicare beneficiary summary files.
Cardiologist care	At least 1 physician evaluation and management outpatient or carrier claim with a specialty code 06 during the look-back period.
Endocrinologist care	At least 1 physician evaluation and management outpatient or carrier claim with a specialty code 46 during the look-back period.
Nephrologist care	At least 1 physician evaluation and management outpatient or carrier claim with a specialty code 39 during the look-back period.
Low income subsidy/Medicare-Medicaid dual eligible	Defined as receipt of any low-income subsidy under Medicare Part D or dual eligible status code of 01-08 or state buy-in entitlement for any month during the look-back period. These codes refer to Medicare beneficiaries for whom Medicaid pays premiums.
Residence area-level median income	Defined using beneficiary's zip code, county, or state of residence listed in the beneficiary summary file on the date of antihypertensive medication initiation and linking to the U.S. Census Bureau's American Community Survey. Median income for the beneficiary's zip code was used if present in the beneficiary summary file. Median income for the beneficiary's county was used if zip code was not present in the beneficiary's summary file. Median income for the beneficiary's state was used if neither zip code nor county was present in the beneficiary's summary file.
Geographical Region of Residence[2]	Defined using beneficiary's state of residence in the beneficiary summary file on the date of antihypertensive medication initiation. States were organized into the following regions as defined by the U.S. Census Bureau:<br>• West South Central: Arkansas, Louisiana, Oklahoma, and Texas.<br>• Mountain: Arizona, Colorado, Idaho, Nevada, New Mexico, Montana, Utah, and Wyoming.<br>• East South Central: Alabama, Kentucky, Mississippi, and Tennessee.<br>• Middle Atlantic: New Jersey, New York, and Pennsylvania.<br>• South Atlantic: Delaware, Maryland, Virginia, West Virginia, North Carolina, South Carolina, Georgia, Florida, and the District of Columbia.<br>• West North Central: Iowa, Kansas, Minnesota, Missouri, Nebraska, North Dakota and South Dakota.<br>• East North Central: Illinois, Indiana, Michigan, Ohio, and Wisconsin.<br>• Pacific: Alaska, California, Hawaii, Oregon, and Washington.<br>• New England: Connecticut, Maine, Massachusetts, New Hampshire, Rhode Island, and Vermont.
Compelling Indications	
Diabetes[3]	Algorithm for diabetes based on ICD-9 codes: Any of the following using all available claims during the look-back period:<br>• At least 1 inpatient claim with a discharge ICD-9 diagnosis code of 250.xx, 357.2, 362.0x, or 366.41 in any position, or<br>• At least 2 evaluation and management outpatient or carrier claims with a ICD-9 diagnosis code of 250.xx, 357.2, 362.0x, or 366.41 in any position occurring at least 7 days apart, or<br>• At least 1 pharmacy claim for an oral antidiabetic drug fill or insulin.

© 2020 Colvin CL et al. *JAMA Network Open*.

Variables	Definition
	Algorithm for diabetes based on ICD-10 codes: Any of the following using all available claims during the look-back period:<br>• At least 1 inpatient claim with a discharge ICD-10 diagnosis code of 'E0836', 'E0842', 'E0936', 'E0942', 'E1010', 'E1011', 'E1029', 'E10311', 'E10319', 'E1036', 'E1039', 'E1040', 'E1042', 'E1051', 'E10618', 'E10620', 'E10621', 'E10622', 'E10628', 'E10630', 'E10638', 'E10641', 'E10649', 'E1065', 'E1069', 'E108', 'E109', 'E1100', 'E1101', 'E1129', 'E11311', 'E11319', 'E11329', 'E11339', 'E11349', 'E11359', 'E1136', 'E1139', 'E1140', 'E1142', 'E1151', 'E11618', 'E11620', 'E11621', 'E11622', 'E11628', 'E11630', 'E11638', 'E11641', 'E11649', 'E1165', 'E1169', 'E118', 'E119', 'E1310', 'E1336', 'E1342' in any position, or<br>• At least 2 evaluation and management outpatient or carrier claims with a ICD-10 diagnosis code of 'E0836', 'E0842', 'E0936', 'E0942', 'E1010', 'E1011', 'E1029', 'E10311', 'E10319', 'E1036', 'E1039', 'E1040', 'E1042', 'E1051', 'E10618', 'E10620', 'E10621', 'E10622', 'E10628', 'E10630', 'E10638', 'E10641', 'E10649', 'E1065', 'E1069', 'E108', 'E109', 'E1100', 'E1101', 'E1129', 'E11311', 'E11319', 'E11329', 'E11339', 'E11349', 'E11359', 'E1136', 'E1139', 'E1140', 'E1142', 'E1151', 'E11618', 'E11620', 'E11621', 'E11622', 'E11628', 'E11630', 'E11638', 'E11641', 'E11649', 'E1165', 'E1169', 'E118', 'E119', 'E1310', 'E1336', 'E1342', in any position occuring at least 7 days apart, or<br>• At least 1 pharmacy claim for an oral antidiabetic drug fill or insulin.
History of Atrial Fibrillation or Flutter	Algorithm based on ICD-9 codes: Any of the following using all available claims during the look-back period:<br>• At least 1 inpatient claim with a discharge ICD-9 diagnosis code of 427.31 427.32 in any position, or<br>• At least 1 outpatient or carrier file claim with ICD-9 diagnosis code of 427.31, 427.32 in any position.<br>Algorithm based on ICD-10 codes: Any of the following using all available claims during the look-back period:<br>• At least 1 inpatient claim with a discharge ICD-10 diagnosis code of I480, I483, I484, I4891, I4892, I481xxx, I482xxx in any position, or<br>• At least 1 outpatient or carrier file claim with ICD-10 diagnosis code of I480, I483, I484, I4891, I4892, I481xxx, I482xxx in any position.
History of Coronary heart disease (CHD)[4,5]	Algorithm based on ICD-9 codes: Any of the following using all available claims during the look-back period:<br>• At least 1 inpatient claim with an ICD-9 diagnosis code of 410.xx-414.xx, V45.81 or V45.82, or<br>• At least 2 outpatient or carrier file claims, linked to E&M code, with ICD-9 diagnosis codes of 410.xx-414.xx, V45.81 or V45.82, with the 2 claims occurring at least 7 days apart, or<br>• An inpatient, outpatient or carrier file claim with an ICD-9 procedure code of 00.66, 36.0, 36.01-36.19, 36.2 or a HCPCS code of 33510-33519, 33521-33523, 33530, 33533-33536, 92980-92982, 92984, 92995, 92996, 92920, 92921, 92924, 92925, 92928, 92929, 92933, 92934, 92937, 92938, 92941, 92943, 92944, 92973, C9600, C9601, C9602, C9603, C9604, C9605, C9606, C9607, C9608, G0290, G0291.<br><br>Algorithm based on ICD-10 codes: Any of the following using all available claims during the look-back period:<br>• At least 1 inpatient claim with an ICD-10 diagnosis code of I200, I201, I208, I209, I21.xx, I22.xx, I240, I241, I248, I2510, I252, I253, I2541, I2542, I255, I25810, I25811, I25812, I2582, I2583, I2584, I2589, I259, 'Z95.1' or 'Z9861', or

© 2020 Colvin CL et al. *JAMA Network Open*.

Variables	Definition
	• At least 2 outpatient or carrier file claims, linked to E&M code, with ICD-10 diagnosis codes I200, I201, I208, I209, I21.xx, I22.xx, I240, I241, I248, I2510, I252, I253, I2541, I2542, I255, I25810, I25811, I25812, I2582, I2583, I2584, I2589, I259, ‘Z95.1’ or ‘Z98.61’, with the 2 claims occurring at least 7 days apart, or<br>• An inpatient, outpatient or carrier file claim with an ICD-10 procedure code of 0210, 0211, 0212, 0213, 0270, 0271, 0272, 0273, 02C0, 02C1, 02C2, 02C3, 3E07 or a HCPCS code of 33510-33519, 33521-33523, 33530, 33533-33536, 92980-92982, 92984, 92995, 92996, 92920, 92921, 92924, 92925, 92928, 92929, 92933, 92934, 92937, 92938, 92941, 92943, 92944, 92973, C9600, C9601, C9602, C9603, C9604, C9605, C9606, C9607, C9608, G0290, G0291.
Stroke[6]	Algorithm based on ICD-9 codes: Any of the following using all available claims during the look-back period:<br>• At least 1 inpatient claim with ICD-9 diagnosis code of 430.xx, 431.xx, 433.x1, 434.x1 or 436.x in a primary or secondary position, or<br>• At least 1 evaluation and management outpatient or carrier claim with ICD-9 diagnose code of 430.xx, 431.xx, 433.x1, 434.x1 or 436.x in any position, or<br>• At least 1 claim with ICD-9 diagnosis code of 430.xx, 431.xx, 433.x1, 434.x1 or 436.x in any position in the Home Health Agency, durable medical equipment, Hospice, or SNF files.<br><br>Algorithm based on ICD-10 codes: Any of the following using all available claims during the look-back period:<br>• At least 1 inpatient claim with ICD-10 diagnosis code of 'I60.xx', 'I61.xx', 'I63.xx', ‘I67.89’, ‘I67850’, ‘I67858’ in a primary or secondary position, or<br>• At least 1 evaluation and management outpatient or carrier claim with ICD-10 diagnose code of 'I60.xx', 'I61.xx', 'I63.xx', ‘I67.89’ in any position, or<br>• At least 1 claim with ICD-10 diagnosis code of 'I60.xx', 'I61.xx', 'I63.xx', ‘I67.89’, ‘I67850’, ‘I67858’ in any position in the Home Health Agency, durable medical equipment, Hospice, or SNF files.
History of chronic kidney disease (CKD)[7,8]	Algorithm based on ICD-9 codes: Any of the following using all available claims during the look-back period (this definition is from the United States Renal Data System [USRDS] annual report):<br>• At least 1 inpatient claim with an ICD-9 discharge diagnosis code of 016.0, 095.4, 189.0, 189.9, 223.0, 236.91, 250.4, 271.4, 274.1, 283.11, 403.x1, 403.x0, 404.x2, 404.x3, 404.x0, 404.x1, 440.1, 442.1, 447.3, 572.4, 580–588, 591, 642.1, 646.2, 753.12–753.17, 753.19, 753.2, 794.4 in any discharge diagnosis position, or<br>• At least 1 physician evaluation and management visit with an ICD-9 diagnosis code of 016.0, 095.4, 189.0, 189.9, 223.0, 236.91, 250.4, 271.4, 274.1, 283.11, 403.x1, 403.x0, 404.x2, 404.x3, 404.x0, 404.x1, 440.1, 442.1, 447.3, 572.4, 580–588, 591, 642.1, 646.2, 753.12–753.17, 753.19, 753.2, or 794.4 in any position, or<br>• If the flag ESRD_IND in the Master beneficiary summary file is checked then the participant will be categorized as having a history of CKD.<br><br>Algorithm based on ICD-10 codes: Any of the following using all available claims during the look-back period:<br>• At least 1 inpatient claim with an ICD-10 discharge diagnosis code of ‘A1811', 'A5275', 'C649', 'C689', 'D3000', 'D4100', 'D4120', 'D593',

Variables	Definition
	'E1021', 'E1029', 'E1121', 'E1129', 'E748', 'I120', 'I129', 'I130', 'I1310', 'I1311', 'I132', I701', 'I722', 'K767', 'M1030', 'N003', 'N008', 'N009', 'N013', 'N022', 'N032', 'N033', 'N035', 'N038', 'N039', 'N040', 'N043', 'N044', 'N048', 'N049', 'N052', 'N055', 'N058', 'N059', 'N08', 'N1330', 'N170', 'N171', 'N172', 'N178', 'N179', 'N181', 'N182', 'N183', 'N184', 'N185', 'N186', 'N189', 'N19', 'N250', 'N251', 'N2581', 'N2589', 'N259', 'N269','Q6102', 'Q6119', 'Q612', 'Q613', 'Q614', 'Q615', 'Q618', 'Q6210', 'Q6211', 'Q6212', 'Q6231', 'Q6239', 'R944' in any discharge diagnosis position, or<br>• At least 1 physician evaluation and management visit with an ICD-10 diagnosis code of 'A1811', 'A5275', 'C649', 'C689', 'D3000', 'D4100', 'D4120', 'D593', 'E1021', 'E1029', 'E1121', 'E1129', 'E748', 'I120', 'I129', 'I130', 'I1310', 'I1311', 'I132', I701', 'I722', 'K767', 'M1030', 'N003', 'N008', 'N009', 'N013', 'N022', 'N032', 'N033', 'N035', 'N038', 'N039', 'N040', 'N043', 'N044', 'N048', 'N049', 'N052', 'N055', 'N058', 'N059', 'N08', 'N1330', 'N170', 'N171', 'N172', 'N178', 'N179', 'N181', 'N182', 'N183', 'N184', 'N185', 'N186', 'N189', 'N19', 'N250', 'N251', 'N2581', 'N2589', 'N259', 'N269','Q6102', 'Q6119', 'Q612', 'Q613', 'Q614', 'Q615', 'Q618', 'Q6210', 'Q6211', 'Q6212', 'Q6231', 'Q6239', 'R944' in any position, or<br>• If the flag ESRD_IND in the Master beneficiary summary file is checked then the participant will be categorized as having a history of CKD.
History of heart failure[9]	Algorithm based on ICD-9 codes: Any of the following using all available claims during the look-back period:<br>• At least 1 hospitalization with an ICD-9 discharge diagnosis code of 402.01, 402.11, 402.91, 404.01, 404.03, 404.11, 404.13, 404.91, 404.93, or 428.x in any discharge diagnosis position, or<br>• At least 2 physician evaluation and management visits with an ICD-9 diagnosis code of 402.01, 402.11, 402.91, 404.01, 404.03, 404.11, 404.13, 404.91, 404.93, or 428.x in any position on separate days.<br>Algorithm based on ICD-10 codes: Any of the following using all available claims during the look-back period:<br>• At least 1 hospitalization with an ICD-10 discharge diagnosis code of 'I110', 'I130', 'I132', 'I501', 'I5020', 'I5021', 'I5022', 'I5023', 'I5030', 'I5031', 'I5032', 'I5033', 'I5040', 'I5041', 'I5042', 'I5043', 'I509', 'I50810', 'I50814', 'I50811', 'I50812', 'I50813', 'I5082', 'I5083', 'I5084', 'I5089' in any discharge position, or<br>• At least 2 physician evaluation and management visits with an ICD-10 diagnosis code of 'I110', 'I130', 'I132', 'I501', 'I5020', 'I5021', 'I5022', 'I5023', 'I5030', 'I5031', 'I5032', 'I5033', 'I5040', 'I5041', 'I5042', 'I5043', 'I509', 'I50810', 'I50814', 'I50811', 'I50812', 'I50813', 'I5082', 'I5083', 'I5084', 'I5089' in any position on separate days.

© 2020 Colvin CL et al. *JAMA Network Open*.

eTable 4. Number and Percentage of Medicare Beneficiaries of Black, White, and Other Races/Ethnicities Initiating Each Class of Antihypertensive Medication, by Calendar Year Among Those Initiating Antihypertensive Medication Monotherapy

	Calendar Year of Antihypertensive Medication Initiation								
**Medication Class**	**2011[a]**	**2012[a]**	**2013[a]**	**2014[a]**	**2015[a]**	**2016[a]**	**2017[a]**	**2018[a]**	**P-trend**
Renin–angiotensin–aldosterone system inhibitor									
Any (ACEI or ARB)									
Black	64 (25.2%)	62 (23.6%)	72 (28.6%)	87 (29.6%)	75 (28.6%)	58 (19.7%)	77 (26.8%)	68 (23.7%)	0.47
White	1,244 (39.4%)	1,253 (39.0%)	1,224 (38.0%)	1,372 (40.8%)	1,327 (39.1%)	1,361 (37.8%)	1,394 (38.8%)	1,556 (41.5%)	0.23
Other	131 (46.1%)	120 (43.5%)	116 (43.0%)	155 (50.2%)	112 (41.8%)	151 (44.8%)	144 (43.1%)	141 (40.8%)	0.22
ACEI									
Black	50 (19.7%)	46 (17.5%)	50 (19.8%)	63 (21.4%)	43 (16.4%)	37 (12.5%)	41 (14.3%)	43 (15.0%)	0.01
White	938 (29.7%)	957 (29.8%)	894 (27.8%)	996 (29.7%)	926 (27.3%)	948 (26.3%)	882 (24.6%)	971 (25.9%)	< 0.001
Other	75 (26.4%)	65 (23.6%)	72 (26.7%)	88 (28.5%)	61 (22.8%)	96 (28.5%)	81 (24.3%)	74 (21.4%)	0.28
ARB									
Black	14 (5.5%)	16 (6.1%)	22 (8.7%)	24 (8.2%)	32 (12.2%)	21 (7.1%)	36 (12.5%)	25 (8.7%)	0.01
White	306 (9.7%)	296 (9.2%)	330 (10.2%)	376 (11.2%)	401 (11.8%)	413 (11.5%)	512 (14.3%)	585 (15.6%)	< 0.001
Other	56 (19.7%)	55 (19.9%)	44 (16.3%)	67 (21.7%)	51 (19.0%)	55 (16.3%)	63 (18.9%)	67 (19.4%)	0.71
Beta-blocker									
Black	36 (14.2%)	43 (16.3%)	39 (15.5%)	36 (12.2%)	35 (13.4%)	41 (13.9%)	34 (11.8%)	32 (11.1%)	0.08
White	635 (20.1%)	681 (21.2%)	654 (20.3%)	661 (19.7%)	652 (19.2%)	690 (19.2%)	620 (17.3%)	576 (15.4%)	< 0.001
Other	32 (11.3%)	35 (12.7%)	56 (20.7%)	46 (14.9%)	44 (16.4%)	59 (17.5%)	46 (13.8%)	52 (15.0%)	0.40
Diuretic									
Thiazide-type									
Black	36 (14.2%)	39 (14.8%)	30 (11.9%)	54 (18.4%)	39 (14.9%)	41 (13.9%)	41 (14.3%)	37 (12.9%)	0.69
White	372 (11.8%)	365 (11.4%)	380 (11.8%)	298 (8.9%)	349 (10.3%)	348 (9.7%)	319 (8.9%)	327 (8.7%)	< 0.001
Other	27 (9.5%)	31 (11.2%)	19 (7.0%)	21 (6.8%)	22 (8.2%)	15 (4.5%)	27 (8.1%)	29 (8.4%)	0.18
Loop									
Black	32 (12.6%)	26 (9.9%)	18 (7.1%)	28 (9.5%)	29 (11.1%)	34 (11.5%)	Redacted[c]	29 (10.1%)	0.68
White	337 (10.7%)	327 (10.2%)	352 (10.9%)	353 (10.5%)	354 (10.4%)	431 (12.0%)	417 (11.6%)	369 (9.9%)	0.54
Other	13 (4.6%)	17 (6.2%)	19 (7.0%)	13 (4.2%)	22 (8.2%)	22 (6.5%)	15 (4.5%)	Redacted[c]	0.67
Potassium-sparing									
Black	Redacted[c]	Redacted[c]	Redacted[c]	Redacted[c]	0 (0.0%)	0 (0.0%)	0 (0.0%)	0 (0.0%)	N/A
White	0 (0.0%)	0 (0.0%)	0 (0.0%)	Redacted[c]	0 (0.0%)	Redacted[c]	0 (0.0%)	Redacted[c]	N/A

© 2020 Colvin CL et al. *JAMA Network Open*.

	Calendar Year of Antihypertensive Medication Initiation								
**Medication Class**	**2011**[a]	**2012**[a]	**2013**[a]	**2014**[a]	**2015**[a]	**2016**[a]	**2017**[a]	**2018**[a]	**P-trend**
Other	0 (0.0%)	0 (0.0%)	Redacted[c]	0 (0.0%)	0 (0.0%)	0 (0.0%)	0 (0.0%)	0 (0.0%)	N/A
Aldosterone receptor antagonist									
Black	Redacted[c]	Redacted[c]	Redacted[c]	Redacted[c]	Redacted[c]	Redacted[c]	Redacted[c]	Redacted[c]	0.78
White	18 (0.6%)	20 (0.6%)	32 (1.0%)	20 (0.6%)	23 (0.7%)	21 (0.6%)	25 (0.7%)	43 (1.1%)	0.08
Other	Redacted[c]	Redacted[c]	Redacted[c]	Redacted[c]	Redacted[c]	0 (0.0%)	Redacted[c]	Redacted[c]	0.73
Calcium channel blocker									
Black	70 (27.6%)	81 (30.8%)	81 (32.1%)	81 (27.6%)	72 (27.5%)	109 (36.9%)	99 (34.5%)	109 (38.0%)	0.003
White	460 (14.6%)	482 (15.0%)	492 (15.3%)	536 (16.0%)	592 (17.4%)	638 (17.7%)	726 (20.2%)	759 (20.3%)	< 0.001
Other	72 (25.4%)	66 (23.9%)	47 (17.4%)	64 (20.7%)	61 (22.8%)	81 (24.0%)	86 (25.7%)	95 (27.5%)	0.13
Other medication class[b]									
Black	13 (5.1%)	Redacted[c]	11 (4.4%)	Redacted[c]	Redacted[c]	Redacted[c]	11 (3.8%)	Redacted[c]	0.46
White	95 (3.0%)	87 (2.7%)	87 (2.7%)	116 (3.5%)	99 (2.9%)	113 (3.1%)	90 (2.5%)	114 (3.0%)	0.99
Other	Redacted[c]	Redacted[c]	12 (4.4%)	Redacted[c]	Redacted[c]	Redacted[c]	14 (4.2%)	12 (3.5%)	0.42

ACEI: Angiotensin-converting enzyme inhibitor, ARB: Angiotensin receptor blocker.

[a] Number of black, white, and other race beneficiaries in each calendar year: **2011**: Black=254, White=3,161, Other race=284; **2012**: Black=263, White=3,215, Other race=276; **2013:** Black=252, White=3,221, Other race=270; **2014:** Black=294, White=3,359, Other race=309; **2015:** Black=262, White=3,396, Other race=268; **2016:** Black=295, White=3,603, Other race=337; **2017:** Black=287, White=3,591, Other race=334; **2018:** Black=287, White=3,745, Other race=346. **Overall:** Black=2,194, White=27,291, Other race=2,424.

[b] Others include alpha blockers, central acting agents, direct vasodilators, and renin inhibitors.

[c] Owing to small numbers.

© 2020 Colvin CL et al. *JAMA Network Open*.

eTable 5. Number and Percentage of Medicare Beneficiaries of Black, White, and Other Races/Ethnicities Initiating Each Class of Antihypertensive Medication, by Calendar Year Among Those Initiating Antihypertensive Combination Therapy

	Calendar Year of Antihypertensive Medication Initiation								
**Medication Class**	**2011[a]**	**2012[a]**	**2013[a]**	**2014[a]**	**2015[a]**	**2016[a]**	**2017[a]**	**2018[a]**	**P-trend**
Renin–angiotensin–aldosterone system inhibitor									
Any (ACEI or ARB)									
Black	93 (72.7%)	119 (73.0%)	105 (70.0%)	107 (69.9%)	96 (74.4%)	85 (63.4%)	93 (69.4%)	85 (72.0%)	0.42
White	719 (68.4%)	727 (69.2%)	691 (69.6%)	709 (72.1%)	670 (70.7%)	653 (72.3%)	600 (70.8%)	602 (68.8%)	0.33
Other	66 (83.5%)	67 (75.3%)	58 (76.3%)	70 (80.5%)	79 (83.2%)	69 (75.8%)	60 (85.7%)	65 (78.3%)	0.81
ACEI									
Black	59 (46.1%)	78 (47.9%)	80 (53.3%)	69 (45.1%)	71 (55.0%)	49 (36.6%)	62 (46.3%)	56 (47.5%)	0.45
White	446 (42.4%)	496 (47.2%)	465 (46.8%)	469 (47.7%)	439 (46.3%)	408 (45.2%)	394 (46.5%)	360 (41.1%)	0.44
Other	39 (49.4%)	32 (36.0%)	34 (44.7%)	39 (44.8%)	41 (43.2%)	33 (36.3%)	31 (44.3%)	32 (38.6%)	0.39
ARB									
Black	35 (27.3%)	43 (26.4%)	26 (17.3%)	39 (25.5%)	25 (19.4%)	37 (27.6%)	31 (23.1%)	29 (24.6%)	0.82
White	275 (26.2%)	238 (22.7%)	230 (23.2%)	242 (24.6%)	238 (25.1%)	248 (27.5%)	211 (24.9%)	249 (28.5%)	0.03
Other	27 (34.2%)	35 (39.3%)	24 (31.6%)	32 (36.8%)	38 (40.0%)	37 (40.7%)	30 (42.9%)	34 (41.0%)	0.19
Beta-blocker									
Black	31 (24.2%)	37 (22.7%)	31 (20.7%)	39 (25.5%)	29 (22.5%)	32 (23.9%)	35 (26.1%)	27 (22.9%)	0.72
White	232 (22.1%)	249 (23.7%)	262 (26.4%)	243 (24.7%)	257 (27.1%)	203 (22.5%)	204 (24.1%)	230 (26.3%)	0.19
Other	16 (20.3%)	26 (29.2%)	17 (22.4%)	23 (26.4%)	14 (14.7%)	24 (26.4%)	15 (21.4%)	15 (18.1%)	0.33
Diuretic									
Thiazide-type									
Black	87 (68.0%)	117 (71.8%)	99 (66.0%)	105 (68.6%)	83 (64.3%)	90 (67.2%)	84 (62.7%)	71 (60.2%)	0.06
White	761 (72.4%)	747 (71.1%)	699 (70.4%)	651 (66.2%)	612 (64.6%)	584 (64.7%)	527 (62.1%)	518 (59.2%)	< 0.001
Other	46 (58.2%)	54 (60.7%)	49 (64.5%)	56 (64.4%)	61 (64.2%)	56 (61.5%)	42 (60.0%)	49 (59.0%)	0.94
Loop									
Black	Redacted[c]	Redacted[c]	Redacted[c]	Redacted[c]	Redacted[c]	Redacted[c]	Redacted[c]	12 (10.2%)	0.63
White	61 (5.8%)	68 (6.5%)	60 (6.0%)	67 (6.8%)	61 (6.4%)	60 (6.6%)	62 (7.3%)	67 (7.7%)	0.08
Other	Redacted[c]	Redacted[c]	Redacted[c]	Redacted[c]	Redacted[c]	Redacted[c]	Redacted[c]	Redacted[c]	0.12
Potassium-sparing									
Black	13 (10.2%)	20 (12.3%)	16 (10.7%)	18 (11.8%)	12 (9.3%)	19 (14.2%)	19 (14.2%)	Redacted[c]	0.83
White	174 (16.6%)	158 (15.0%)	130 (13.1%)	110 (11.2%)	135 (14.2%)	130 (14.4%)	110 (13.0%)	98 (11.2%)	0.004

© 2020 Colvin CL et al. *JAMA Network Open*.

	Calendar Year of Antihypertensive Medication Initiation								
**Medication Class**	**2011[a]**	**2012[a]**	**2013[a]**	**2014[a]**	**2015[a]**	**2016[a]**	**2017[a]**	**2018[a]**	**P-trend**
Other	Redacted[c]	Redacted[c]	Redacted[c]	Redacted[c]	Redacted[c]	Redacted[c]	Redacted[c]	Redacted[c]	0.75
Aldosterone receptor antagonist									
Black	Redacted[c]	Redacted[c]	Redacted[c]	Redacted[c]	0 (0.0%)	Redacted[c]	Redacted[c]	Redacted[c]	0.34
White	11 (1.0%)	11 (1.0%)	15 (1.5%)	16 (1.6%)	16 (1.7%)	25 (2.8%)	20 (2.4%)	19 (2.2%)	< 0.001
Other	Redacted[c]	Redacted[c]	Redacted[c]	Redacted[c]	Redacted[c]	Redacted[c]	Redacted[c]	Redacted[c]	0.81
Calcium channel blocker									
Black	45 (35.2%)	58 (35.6%)	59 (39.3%)	59 (38.6%)	46 (35.7%)	59 (44.0%)	60 (44.8%)	45 (38.1%)	0.13
White	228 (21.7%)	222 (21.1%)	236 (23.8%)	245 (24.9%)	236 (24.9%)	226 (25.0%)	233 (27.5%)	245 (28.0%)	< 0.001
Other	29 (36.7%)	35 (39.3%)	27 (35.5%)	29 (33.3%)	32 (33.7%)	31 (34.1%)	16 (22.9%)	34 (41.0%)	0.48
Other medication class[b]									
Black	Redacted[c]	Redacted[c]	14 (9.3%)	Redacted[c]	Redacted[c]	11 (8.2%)	Redacted[c]	Redacted[c]	0.29
White	54 (5.1%)	44 (4.2%)	37 (3.7%)	43 (4.4%)	50 (5.3%)	35 (3.9%)	46 (5.4%)	59 (6.7%)	0.06
Other	Redacted[c]	Redacted[c]	Redacted[c]	Redacted[c]	Redacted[c]	Redacted[c]	Redacted[c]	Redacted[c]	0.25

ACEI: Angiotensin-converting enzyme inhibitor, ARB: Angiotensin receptor blocker.
[a] Number of black, white, and other race beneficiaries in each calendar year: **2011**: Black=128, White=1,051, Other race=79; **2012**: Black=163, White=1,050, Other race=89; **2013:** Black=150, White=993, Other race=76; **2014:** Black=153, White=984, Other race=87; **2015:** Black=129, White=948, Other race=95; **2016:** Black=134, White=903, Other race=91; **2017:** Black=134, White=848, Other race=70; **2018:** Black=118, White=875, Other race=83. Overall: Black=1,109, White=7,652, Other race=670.
[b] Others include alpha blockers, central acting agents, direct vasodilators, and renin inhibitors.
[c] Owing to small numbers.

© 2020 Colvin CL et al. *JAMA Network Open*.

eTable 6. Number and Percentage of Medicare Beneficiaries of Black, White, and Other Races/Ethnicities Initiating the Most Common Combinations of Dual and Triple Therapies, by Calendar Year Among Those Initiating Two or More Classes of Antihypertensive Medication

**BLACKS**	**Calendar Year of Antihypertensive Medication Initiation**								
**Antihypertensive medication class combinations**	**2011 n=128**	**2012 n=163**	**2013 n=150**	**2014 n=153**	**2015 n=129**	**2016 n=134**	**2017 n=134**	**2018 n=118**	**P-trend**
Dual Therapy									
1.ACEI+ thiazide-type	23 (18.0%)	35 (21.5%)	42 (28.0%)	33 (21.6%)	35 (27.1%)	21 (15.7%)	27 (20.1%)	20 (16.9%)	0.30
2.Thiazide-type+ARBs	21 (16.4%)	26 (16.0%)	11 (7.3%)	20 (13.1%)	14 (10.9%)	20 (14.9%)	12 (9.0%)	17 (14.4%)	0.43
3.Thiazide-type+potassium sparing	12 (9.4%)	12 (7.4%)	11 (7.3%)	13 (8.5%)	12 (9.3%)	14 (10.4%)	13 (9.7%)	Redacted[a]	0.57
4.ACEI+CCB	12 (9.4%)	17 (10.4%)	Redacted[a]	12 (7.8%)	13 (10.1%)	Redacted[a]	16 (11.9%)	12 (10.2%)	0.62
Triple Therapy									
1.ACEI+ thiazide-type+CCB	Redacted[a]	Redacted[a]	Redacted[a]	Redacted[a]	Redacted[a]	Redacted[a]	Redacted[a]	Redacted[a]	0.45
2.Thiazide-type+ARBs+CCB	Redacted[a]	Redacted[a]	Redacted[a]	Redacted[a]	Redacted[a]	Redacted[a]	Redacted[a]	Redacted[a]	0.69
3.Thiazide-type+ACEI+beta-blocker	Redacted[a]	Redacted[a]	Redacted[a]	Redacted[a]	Redacted[a]	Redacted[a]	Redacted[a]	Redacted[a]	0.54
4.ACEI+CCB+beta-blocker	Redacted[a]	0 (0.0%)	0 (0.0%)	Redacted[a]	Redacted[a]	Redacted[a]	Redacted[a]	0 (0.0%)	0.48
**WHITES**		**Calendar Year of Antihypertensive Medication Initiation**							
**Antihypertensive medication class combinations**	**2011 n=1,051**	**2012 n=1050**	**2013 n=993**	**2014 n=984**	**2015 n=948**	**2016 n=903**	**2017 n=848**	**2018 n=875**	**P-trend**
Dual Therapy									
1.ACEI+ thiazide-type	253 (24.1%)	279 (26.6%)	255 (25.7%)	249 (25.3%)	194 (20.5%)	192 (21.3%)	182 (21.5%)	169 (19.3%)	< 0.001
2.Thiazide-type+ARBs	179 (17.0%)	159 (15.1%)	146 (14.7%)	150 (15.2%)	144 (15.2%)	151 (16.7%)	123 (14.5%)	142 (16.2%)	0.83
3.Thiazide-type+potassium sparing	150 (14.3%)	139 (13.2%)	107 (10.8%)	90 (9.1%)	114 (12.0%)	119 (13.2%)	102 (12.0%)	86 (9.8%)	0.04
4.ACEI+CCB	57 (5.4%)	74 (7.0%)	71 (7.2%)	75 (7.6%)	80 (8.4%)	75 (8.3%)	79 (9.3%)	79 (9.0%)	< 0.001
Triple Therapy									
1.ACEI+ thiazide-type+CCB	15 (1.4%)	Redacted[a]	14 (1.4%)	19 (1.9%)	Redacted[a]	14 (1.6%)	13 (1.5%)	Redacted[a]	0.90
2.Thiazide-type+ARBs+CCB	16 (1.5%)	13 (1.2%)	Redacted[a]	Redacted[a]	11 (1.2%)	14 (1.6%)	Redacted[a]	Redacted[a]	0.44
3.Thiazide-type+ACEI+beta-blocker	Redacted[a]	22 (2.1%)	19 (1.9%)	11 (1.1%)	22 (2.3%)	Redacted[a]	15 (1.8%)	Redacted[a]	0.14
4.ACEI+CCB+beta-blocker	Redacted[a]	Redacted[a]	Redacted[a]	Redacted[a]	Redacted[a]	Redacted[a]	Redacted[a]	Redacted[a]	0.11
**Other Races/Ethnicities**		**Calendar Year of Antihypertensive Medication Initiation**							

© 2020 Colvin CL et al. *JAMA Network Open*.

Antihypertensive medication class combinations	2011 n=79	2012 n=89	2013 n=76	2014 n=87	2015 n=95	2016 n=91	2017 n=70	2018 n=83	P-trend
Dual Therapy									
1.ACEI+ thiazide-type	16 (20.3%)	12 (13.5%)	18 (23.7%)	20 (23.0%)	25 (26.3%)	13 (14.3%)	11 (15.7%)	15 (18.1%)	0.67
2.Thiazide-type+ARBs	15 (19.0%)	20 (22.5%)	15 (19.7%)	17 (19.5%)	20 (21.1%)	25 (27.5%)	18 (25.7%)	20 (24.1%)	0.21
3.Thiazide-type+potassium sparing	Redacted[a]	Redacted[a]	Redacted[a]	Redacted[a]	Redacted[a]	Redacted[a]	Redacted[a]	Redacted[a]	0.55
4.ACEI+CCB	14 (17.7%)	Redacted[a]	Redacted[a]	Redacted[a]	Redacted[a]	Redacted[a]	Redacted[a]	Redacted[a]	0.45
Triple Therapy									
1.ACEI+ thiazide-type+CCB	0 (0.0%)	Redacted[a]	0 (0.0%)	Redacted[a]	Redacted[a]	Redacted[a]	0 (0.0%)	Redacted[a]	0.87
2.Thiazide-type+ARBs+CCB	Redacted[a]	Redacted[a]	0 (0.0%)	Redacted[a]	Redacted[a]	Redacted[a]	Redacted[a]	Redacted[a]	0.99
3.Thiazide-type+ACEI+beta-blocker	Redacted[a]	Redacted[a]	Redacted[a]	Redacted[a]	0 (0.0%)	Redacted[a]	Redacted[a]	0 (0.0%)	0.42
4.ACEI+CCB+beta-blocker	Redacted[a]	Redacted[a]	Redacted[a]	Redacted[a]	Redacted[a]	Redacted[a]	0 (0.0%)	Redacted[a]	0.80

CCB: Calcium channel blocker, ACEI: Angiotensin-converting enzyme inhibitor, ARB: Angiotensin receptor blocker.
[a] Owing to small numbers.

© 2020 Colvin CL et al. *JAMA Network Open*.

eFigure 1. Flowchart for Determining the Eligible Study Population

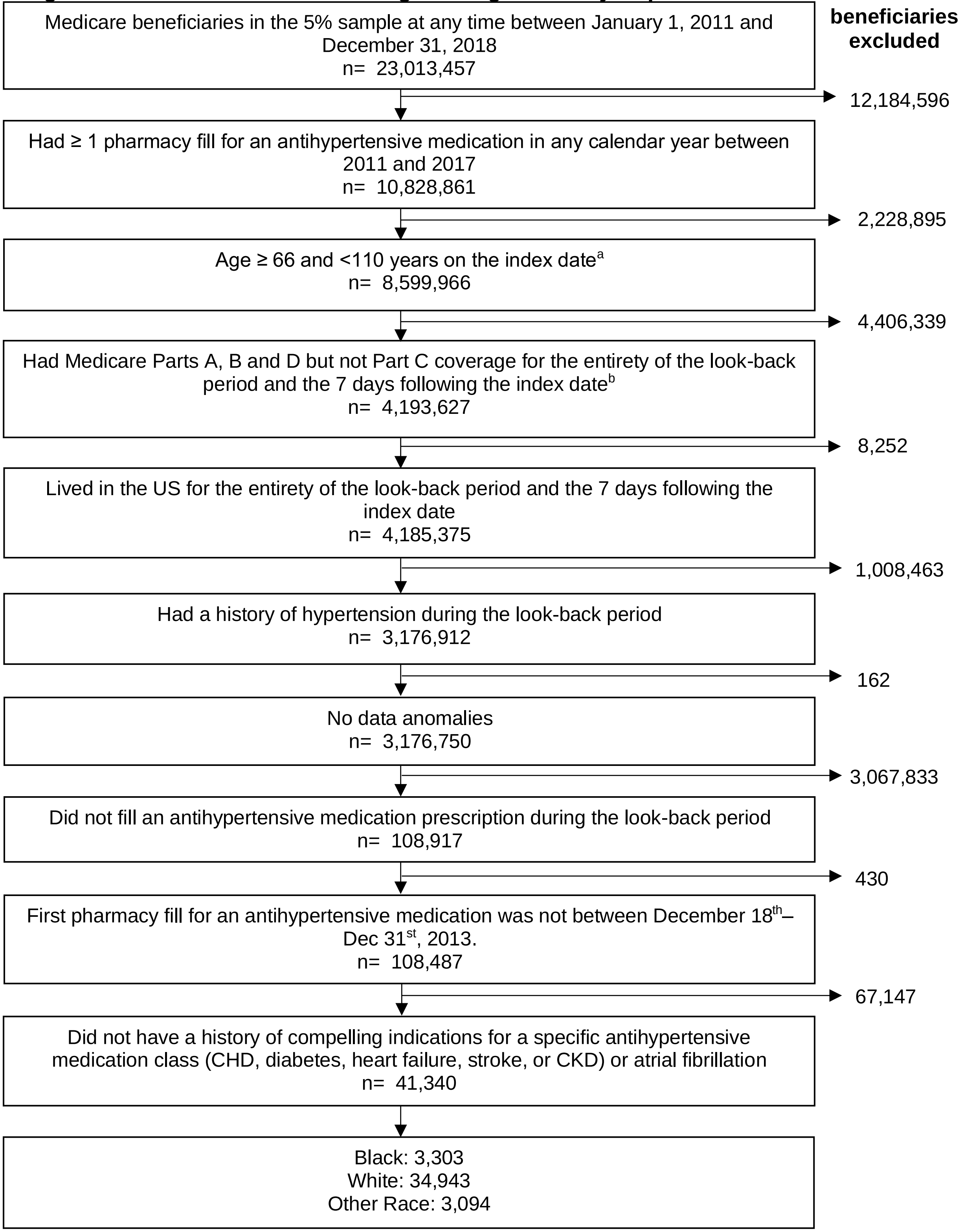

CHD: Coronary heart disease, CKD: Chronic kidney disease.

[a] The date of beneficiaries' first antihypertensive medication fill in each calendar defined their index date.

[b] The 365 days preceding the index date defined the look-back period.

© 2020 Colvin CL et al. *JAMA Network Open*.

eFigure 2. Proportion of Medicare Beneficiaries of Black, White, and Other Races/Ethnicities Initiating One, Two, Three, or Four or More Antihypertensive Medication Classes by Calendar Year

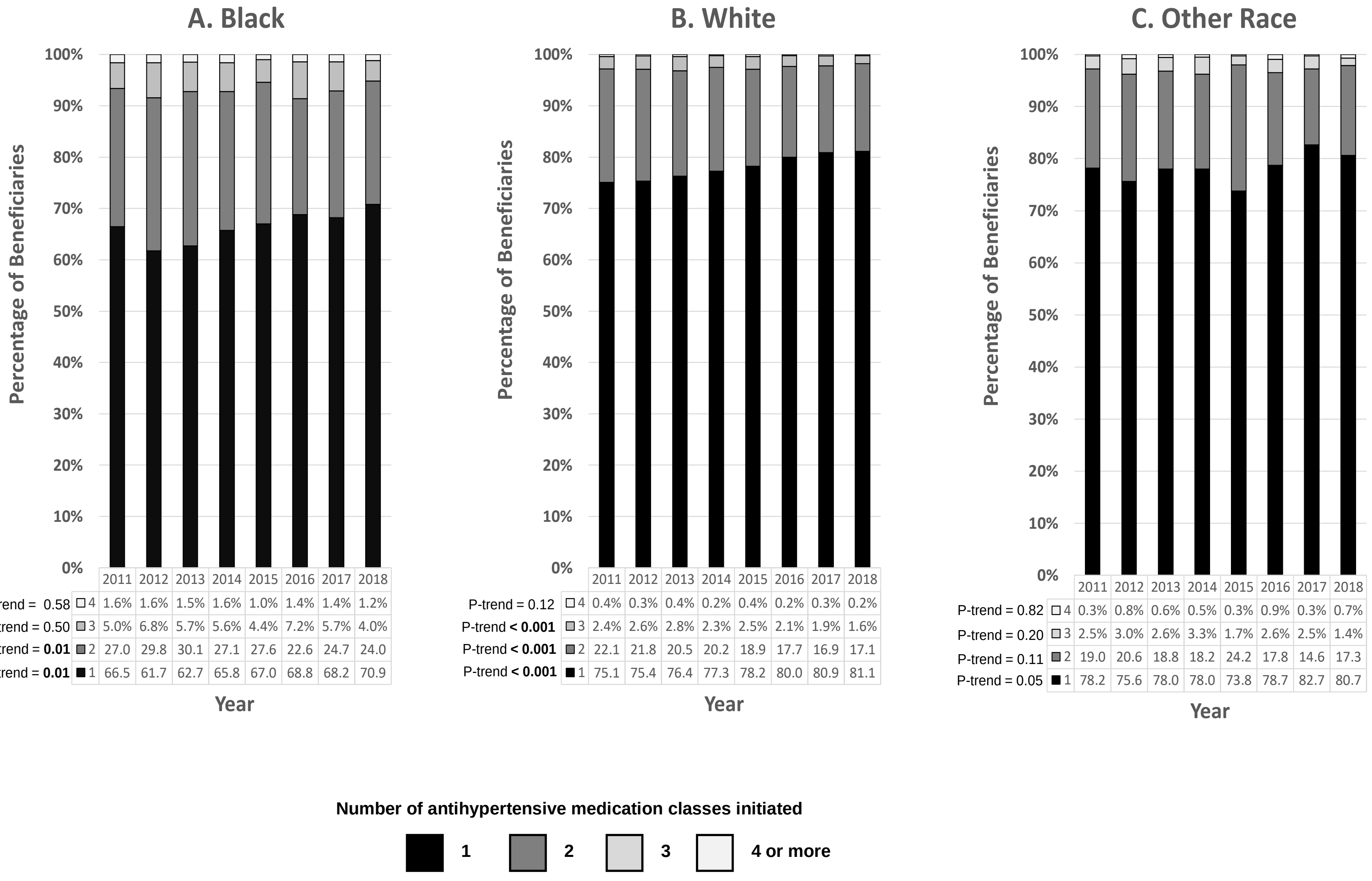

A. Black

P-trend	Classes	2011	2012	2013	2014	2015	2016	2017	2018
P-trend = 0.58	4	1.6%	1.6%	1.5%	1.6%	1.0%	1.4%	1.4%	1.2%
P-trend = 0.50	3	5.0%	6.8%	5.7%	5.6%	4.4%	7.2%	5.7%	4.0%
P-trend = **0.01**	2	27.0	29.8	30.1	27.1	27.6	22.6	24.7	24.0
P-trend = **0.01**	1	66.5	61.7	62.7	65.8	67.0	68.8	68.2	70.9

B. White

P-trend	Classes	2011	2012	2013	2014	2015	2016	2017	2018
P-trend = 0.12	4	0.4%	0.3%	0.4%	0.2%	0.4%	0.2%	0.3%	0.2%
P-trend **< 0.001**	3	2.4%	2.6%	2.8%	2.3%	2.5%	2.1%	1.9%	1.6%
P-trend **< 0.001**	2	22.1	21.8	20.5	20.2	18.9	17.7	16.9	17.1
P-trend **< 0.001**	1	75.1	75.4	76.4	77.3	78.2	80.0	80.9	81.1

C. Other Race

P-trend	Classes	2011	2012	2013	2014	2015	2016	2017	2018
P-trend = 0.82	4	0.3%	0.8%	0.6%	0.5%	0.3%	0.9%	0.3%	0.7%
P-trend = 0.20	3	2.5%	3.0%	2.6%	3.3%	1.7%	2.6%	2.5%	1.4%
P-trend = 0.11	2	19.0	20.6	18.8	18.2	24.2	17.8	14.6	17.3
P-trend = 0.05	1	78.2	75.6	78.0	78.0	73.8	78.7	82.7	80.7

© 2020 Colvin CL et al. *JAMA Network Open*.

eReferences

1. Chobanian AV, Bakris GL, Black HR, et al. The Seventh Report of the Joint National Committee on Prevention, Detection, Evaluation, and Treatment of High Blood PressureThe JNC 7 Report. *JAMA.* 2003;289(19):2560-2571.
2. 2010 Census Regions and Divisions of the United States. United States Census Bureau website. https://www.census.gov/geographies/reference-maps/2010/geo/2010-census-regions-and-divisions-of-the-united-states.html. Updated August 20, 2018. Accessed August 20, 2020.
3. Gandra SR, Lawrence LW, Parasuraman BM, Darin RM, Sherman JJ, Wall JL. Total and component health care costs in a non-Medicare HMO population of patients with and without type 2 diabetes and with and without macrovascular disease. *J Manag Care Pharm.* 2006;12(7):546-554.
4. Culler SD, Kugelmass AD, Brown PP, Reynolds MR, Simon AW. Trends in coronary revascularization procedures among Medicare beneficiaries between 2008 and 2012. *Circulation.* 2015;131(4):362-370; discussion 370.
5. Cutrona SL, Toh S, Iyer A, et al. Design for validation of acute myocardial infarction cases in Mini-Sentinel. *Pharmacoepidemiol Drug Saf.* 2012;21 Suppl 1(0 1):274-281.
6. Kumamaru H, Judd SE, Curtis JR, et al. Validity of claims-based stroke algorithms in contemporary Medicare data: reasons for geographic and racial differences in stroke (REGARDS) study linked with medicare claims. *Circ Cardiovasc Qual Outcomes.* 2014;7(4):611-619.

© 2020 Colvin CL et al. *JAMA Network Open*.

7. Vlasschaert ME, Bejaimal SA, Hackam DG, et al. Validity of administrative database coding for kidney disease: a systematic review. *Am J Kidney Dis.* 2011;57(1):29-43.

8. Muntner P, Gutierrez OM, Zhao H, et al. Validation study of medicare claims to identify older US adults with CKD using the Reasons for Geographic and Racial Differences in Stroke (REGARDS) Study. *Am J Kidney Dis.* 2015;65(2):249-258.

9. Schneider KM, O'Donnell BE, Dean D. Prevalence of multiple chronic conditions in the United States' Medicare population. *Health Qual Life Outcomes.* 2009;7:82.

© 2020 Colvin CL et al. *JAMA Network Open*.
